# Strategies and applications for alleviating carbon catabolite repression

**DOI:** 10.1016/j.synbio.2026.08.005

**Published:** 2026-08-20

**Authors:** Mengtian Lu, Haoran Ding, Hongyu Zhang, Jianyu Xu, Ru Meng, Yihan Li, Zhidan Zhang, Ping Zheng, Yan Zhu, Jibin Sun

**Affiliations:** a Graduate College, Tianjin University of Traditional Chinese Medicine, Tianjin, 301617, China; b Tianjin Institute of Industrial Biotechnology, Chinese Academy of Sciences, Tianjin, 300308, China; c University of Chinese Academy of Sciences, Beijing, 100049, China; d State Key Laboratory of Engineering Biology for Low-Carbon Manufacturing, Tianjin Institute of Industrial Biotechnology, Chinese Academy of Sciences, Tianjin, 300308, China; e National Center of Technology Innovation for Synthetic Biology, Tianjin, 300308, China

**Keywords:** Carbon catabolite repression, Adaptive laboratory evolution, Synthetic biology, Systems biology, Mixed-carbon-source utilisation

## Abstract

Carbon catabolite repression (CCR) is a widespread regulatory strategy across diverse microorganisms that prioritises the utilisation of preferred carbon sources. In industrial bioprocesses, however, CCR-mediated substrate hierarchy can delay the utilisation of secondary carbon sources in mixed feedstock, thereby extending fermentation time and limiting carbon conversion efficiency toward target products. Existing reviews have largely addressed CCR in a microorganism-specific manner, potentially obscuring conserved principles, limiting cross-species comparisons, and constraining generalisable engineering; computational modelling and engineering applications also remain underrepresented. This review therefore first summarises CCR mechanisms across diverse microorganisms through a common systems-level framework, in which transport-linked sensing converts carbon flux into intracellular signals, transcriptional regulators reprogramme genome-wide expression, RNA-based mechanisms fine-tune the timing and magnitude of responses, and protein stability shapes their persistence. Multi-omics approaches, particularly interactomics and single-cell omics, are then highlighted for revealing CCR as a dynamic and multilayered regulatory system by resolving interaction networks, temporal changes, and cell-to-cell heterogeneity beyond bulk measurements. Quantitative computational modelling is also discussed, with emphasis on kinetic and constraint-based frameworks that connect regulatory mechanisms to metabolic flux and predict system behaviour under changing carbon substrates. Engineering strategies, including adaptive laboratory evolution, transcription factor engineering, transporter and promoter engineering, and pathway rewiring, are further compared for the relief of CCR and improvement of substrate co-utilisation and production performance. Finally, these advances are presented within a Design-Build-Test-Learn framework, through which mechanistic discovery and omics-scale analysis are used to inform model development, and modelling and engineering are iteratively coupled to improve microbial performance. We propose that such an integrative paradigm is emerging as a unifying direction for CCR research, and provides a foundation for rational strain design and more efficient, sustainable biomanufacturing.

## 1 Introduction

In natural ecosystems, microorganisms are typically exposed to mixtures of carbon sources rather than a single, defined nutrient. Across metabolically versatile microorganisms, CCR describes a broadly conserved phenotypic output of preferential substrate utilisation, whereby preferred substrates, often glucose in many well-characterised bacterial and yeast systems, suppress or inhibit the transport and catabolism of alternative substrates (e.g. xylose, arabinose, cellobiose, glycerol and acetate) [1,2]. CCR is widespread across metabolically versatile bacteria, yeasts and filamentous fungi, and operates as a coordinated multi-layered regulatory network, rather than a single control mechanism. It integrates metabolic flux sensing via intracellular effectors and key metabolites (e.g. cAMP, glycolytic intermediates), transcriptional regulation by global regulators (e.g. CRP, CcpA, CreA, Mlc), sRNA-mediated control (e.g. CrcZ/CrcY), and post-translational regulation of key transporters and enzymes (e.g. phosphoenolpyruvate:sugar phosphotransferase systems, adenylyl cyclase) [1,3]. Functionally, CCR acts as a global resource allocation module by repressing unnecessary transporters and catabolic enzymes, thereby reducing futile protein synthesis, improving proteome economy, and maximising growth efficiency under nutrient-rich conditions [1]. In this sense, CCR is not intrinsically inefficient; rather, it represents an evolutionarily favourable strategy for prioritising nutrient use and avoiding unnecessary expression of competing catabolic systems. The engineering challenge arises when this fitness-oriented substrate hierarchy conflicts with production objectives that require rapid and balanced conversion of defined mixed-carbon feedstocks. Carbon sensing is also tightly coupled with broader cellular processes, including stress responses, persister formation, secondary metabolism and virulence regulation, linking nutrient status to survival and competitiveness in ever-changing environments [1,3].

In industrial processes, however, CCR becomes a major constraint on the utilisation of renewable feedstocks. Lignocellulosic biomass is estimated to exceed 181.5 billion tonnes per year, and is primarily composed of cellulose and hemicellulose, which together typically account for 60-80% of its dry weight [4,5]. Pretreatment and hydrolysis of agricultural residues and forestry byproducts yield mixed-sugar streams that are typically dominated by glucose (∼30–50%), xylose (∼20–30%), with smaller amounts of arabinose (∼5–10%) and minor fractions of mannose, galactose and cellobiose, depending on feedstock and processing conditions [6,7]. These hydrolysates, along with other low-cost mixed substrates widely used in industrial fermentation (e.g. molasses, corn steep liquor, soybean meal), represent attractive feedstocks for biomanufacturing [4,6]. Yet many industrial strains such as *Escherichia coli*, *Bacillus subtilis*, and *Pseudomonas putida* exhibit CCR-driven diauxic growth under these conditions [6,8,9]. Under such mixed-carbon conditions, many industrial strains first consume glucose and delay the utilisation of non-preferred sugars such as xylose and arabinose. The transition from glucose metabolism to secondary-substrate utilisation can introduce a diauxic lag, during which growth and product formation may slow down. From a bioprocess perspective, this delayed conversion of secondary substrates can prolong fermentation, reduce volumetric productivity and limit the overall conversion of mixed feedstocks into target products. Therefore, the engineering goal is not to force cells to metabolise all available substrates indiscriminately, but to achieve balanced and production-oriented utilisation of selected carbon sources under defined process conditions [1,6].

Despite decades of research on CCR, many mechanistic questions remain unresolved, particularly how multilayered networks give rise to strain-dependent differences in substrate preference, co-utilisation, and growth behaviour under mixed-substrate conditions. In addition, bulk measurements often mask single-cell heterogeneity, even when overall growth appears similar. Recent temporal multi-omics and single-cell analyses are reshaping the study of CCR by revealing dynamic state transitions, subpopulation-specific behaviours, and regulatory trade-offs that are invisible in steady-state or population-averaged datasets. Furthermore, modelling approaches are increasingly employed in CCR studies, with kinetic models capturing sequential substrate uptake, regulator-mediated switching, and metabolite-dependent feedback over time, and constraint-based models analysing how CCR reshapes feasible flux states through changes in carbon allocation, proteome investment, and enzyme-constrained resource reallocation at genome scale.

Although CCR has been extensively reviewed, most existing discussions are organised around specific microorganisms, regulators, or pathways, and therefore mainly emphasise individual mechanisms [1,3]. Yet CCR research now extends beyond mechanistic analysis to systems-level profiling, quantitative modelling, and engineering, making a Design-Build-Test-Learn (DBTL) framework useful for linking discovery to prediction and application [10]. This review is therefore structured to (i) summarise established molecular mechanisms and recent systems-level discoveries, (ii) compare computational modelling approaches for analysing CCR dynamics and substrate utilisation, (iii) evaluate engineering strategies for constructing CCR-resistant or CCR-reprogrammed microbial chassis for efficient co-utilisation of mixed carbon sources, and (iv) highlight emerging efforts to repurpose CCR as a programmable sensing and regulatory module for synthetic biology and sustainable biomanufacturing.

## 2 Systems-level understanding of CCR mechanisms

**Fig. 1:**
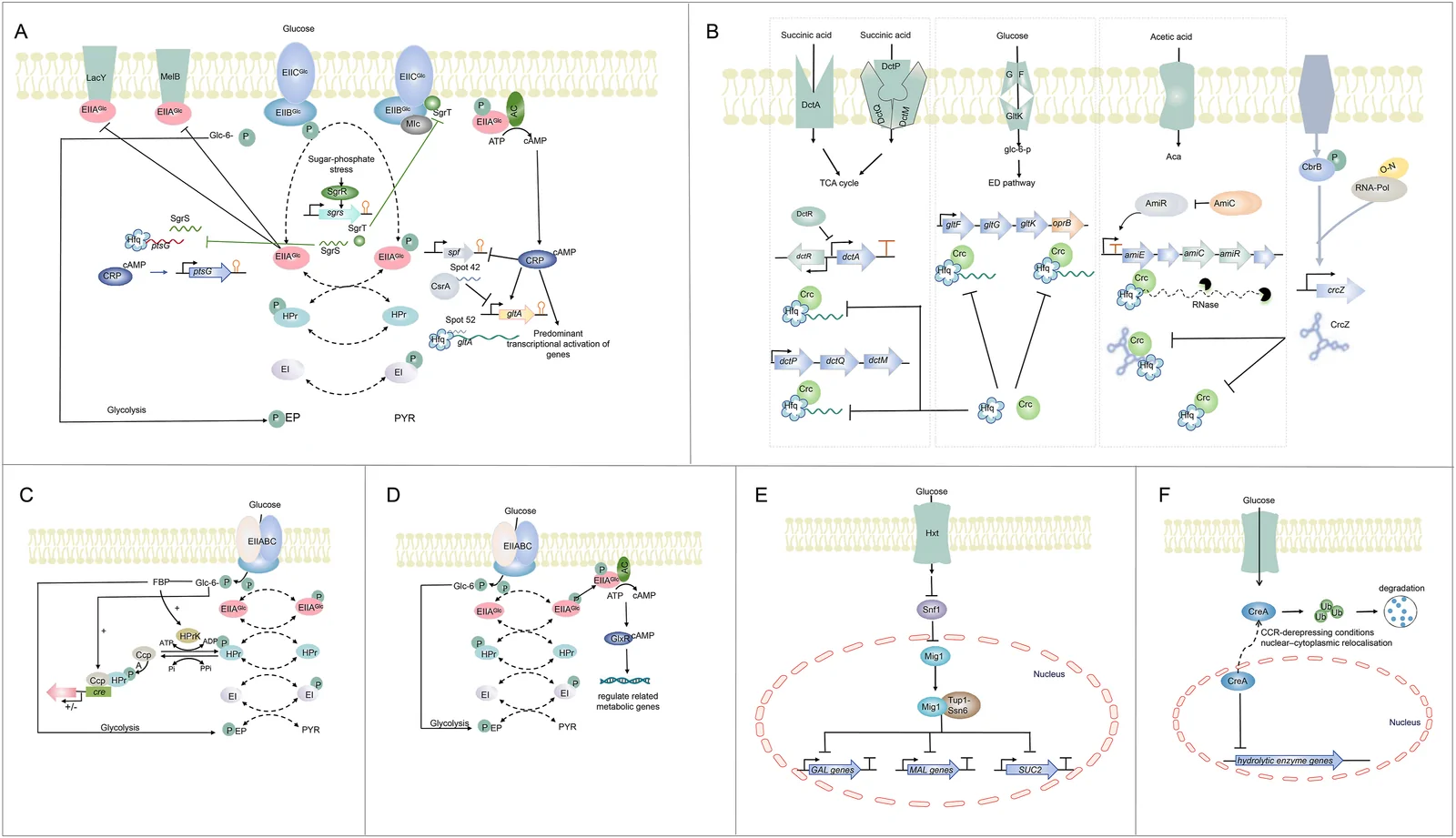
**Comparative mechanisms of carbon catabolite repression (CCR) in representative microorganisms.** (A) *Escherichia coli*. CCR is primarily mediated by the glucose-specific phosphotransferase system (PTS), where the phosphorylation state of EIIA^Glc^ links glucose uptake to transcriptional repression and inducer exclusion. During glucose utilisation, dephosphorylated EIIA^Glc^ inhibits non-PTS transporters and adenylate cyclase, reducing cAMP-CRP-dependent activation of alternative-carbon operons. Hfq-associated sRNAs, including Spot 42 and SgrS, provide additional post-translational regulation. (**B**) *Pseudomonas aeruginosa*. CCR operates predominantly at the post-transcriptional level via the Hfq-Crc system, with CrcZ/CrcY sRNAs modulating repression in response to metabolic status sensed by the CbrA-CbrB system. (**C**) *Bacillus subtilis*. CCR is controlled by the HPrK/P–HPr-Ser46-P–CcpA pathway. In response to glycolytic intermediates and high-energy status, HPrK/P phosphorylates HPr at Ser46; HPr-Ser46-P (or in some contexts Crh-Ser46-P) promotes CcpA binding to catabolite-responsive elements (*cre*), repressing or activating genes involved in secondary-carbon metabolism and overflow pathways. (**D**) *Corynebacterium glutamicum*. CCR is comparatively weak and non-canonical. Carbon-source-dependent regulation is largely mediated by the cAMP-responsive global regulator GlxR, together with sugar- and PTS-associated regulators such as SugR. Unlike classical PTS-driven inducer exclusion, this network primarily reshapes transcription of sugar uptake, glycolysis, respiration, and amino-acid metabolism, enabling flexible carbon-source co-utilisation. (**E**) *Saccharomyces cerevisiae*. CCR is mainly mediated by the glucose-responsive Snf1-Mig1-Tup 1/Ssn6 regulatory axis. Under glucose-repressive conditions, glucose uptake through Hxt transporters is associated with Snf1 inactivation, preventing Snf1-dependent phosphorylation of Mig1. Dephosphorylated Mig1 remains in the nucleus and recruits the Tup1/Ssn6 co-repressor complex to promoters of alternative-carbon utilisation genes, including *GAL* genes, *MAL* genes, and *SUC2*, thereby suppressing their transcription during glucose utilisation. (**F**) *Aspergillus nidulans*. CCR is mediated by CreA-dependent transcriptional repression together with post-translational control of CreA stability and subcellular localisation. Under glucose-repressive conditions, CreA accumulates in the nucleus and represses genes involved in alternative-carbon utilisation and plant-polysaccharide degradation. Under CCR-derepressing conditions, CreA undergoes nuclear-to-cytoplasmic relocalisation, followed by ubiquitination and degradation in the cytoplasm, thereby relieving CreA-dependent repression and enabling expression of hydrolytic enzyme genes.

From bacteria to fungi, CCR functions as a multi-layered regulatory architecture that coordinates carbon transport and sensing, transcriptional hierarchies, RNA-level control, and regulator stability to optimise resource allocation. Although the underlying molecular components differ across taxa, a common systems-level logic emerges that maximises growth efficiency, minimises unnecessary expenditures on enzyme synthesis, and prioritises the rapid utilisation of preferred substrates (Table 1, Fig. 1).

**Table 1:** Comparative summary of CCR mechanisms, regulatory factors, and representative targets across microbial species.

Microorganism	Dominant CCR mechanism	Key regulatory molecule(s)/signal(s)	Major regulatory targets and pathways	References
*Escherichia coli*	PTS-dependent regulation integrating transcriptional control and inducer exclusion	CRP-cAMP, phosphorylation state of EIIA^Glc^, CyaA, PTS flux	cAMP–CRP-dependent operons for secondary-carbon use, including *lacZYA*, *araBAD*, *malEFG*/*malK*, *glpFK*, *rhaBAD*, *xylAB*, and *mglBAC*; inducer exclusion through inhibition of permeases/enzymes (e.g. LacY, MalK, and GlpK); modulation of glyoxylate/TCA-associated genes (e.g. *aceBAK*)	[1] [3] [11]
*Bacillus subtilis*	CcpA-dependent transcriptional repression/activation through HPr/Crh Ser46 phosphorylation	HPr-Ser46-P, Crh-Ser46-P, HPrK/P, CcpA, FBP, G6P, *cre* sites	*cre*-containing genes for alternative-carbon uptake and catabolism, including *xyl*, *ara*, *gnt*, *iol*; overflow metabolism and redox balancing, including *alsSD*, and *ackA-pta*	[1] [3] [8]
*Saccharomyces cerevisiae*	Glucose repression through kinase signalling, transcriptional repression, glucose sensing, and chromatin-level repression	Mig1/Mig2/Mig3, Tup1-Ssn6/Cyc8, Reg1-Glc7, Snf1/AMPK, Snf3/Rgt2, Mth1/Std1, Grr1	Repression of alternative-carbon and respiratory genes, including *GAL1/7/10*, *SUC2*, *MAL*, FBP1, *PCK1*; glucose-dependent control of hexose transporters (e.g. HXT2*/6/7*)	[12] [13] [14]
*Aspergillus nidulans*	CreA-mediated transcriptional repression integrating carbon sensing, promoter-level regulation, and post-translational control	CreA (or Cre1), CreB-CreC	Repression of alternative carbon utilisation regulons, including ethanol (*alc*), proline (*prn*), xylose (*xln*), starch (*amy*), and plant polysaccharide degradation genes	[3,15]
*Pseudomonas* spp.	Post-transcriptional CCR, favouring organic acids and amino acids over sugars	CbrA/CbrB, Crc-Hfq, CrcZ/CrcY sRNAs, RpoN	Hfq/Crc-mediated translational repression of less-preferred catabolic mRNAs, including pathways for glucose, aromatic compounds, alkanes/amides, amino acids, and organic acids; representative genes include *oprB*, *amiE*, *alkS*, *ben*, *ant*, and branched-chain amino-acid catabolic genes	[3] [16] [17] [[18], [19], [20]]
*Corynebacterium glutamicum*	Non-canonical and relatively weak CCR; cAMP-dependent dual-mode transcriptional regulation (activation/repression)	GlxR-cAMP, SugR, RamA, RamB, GntR-family regulators, sugar phosphates (e.g. F1P, FBP)	Sugar uptake and PTS genes (*ptsG*, *iolT1*), glycolysis (*pfkA*, *gapA-pgk-tpi*, *pyk*), TCA and respiration (*sdhCAB*, *gltA*), amino acid biosynthesis	[3] [21] [22]
*Lactobacillus* spp.	CcpA-centred CCR coupled to HPrK/P-dependent HPr Ser46 phosphorylation and species-specific PTS control	HPr-Ser-P, CcpA, HPrK/P, cre sites, FBP, PTS components	Carbohydrate utilisation hierarchy, NADH reoxidation and lactic fermentation	[[23], [24], [25]]
*Streptococcus* spp.	CcpA-dependent CCR plus prominent CcpA-independent PTS/HPr-mediated control in several species	HPr-Ser-P, CcpA, HPrK/P	Sugar metabolism, lactic fermentation, virulence and biofilm-associated genes	[26] [27] [28]
*Streptomyces* spp.	Multi-layer metabolite-responsive regulation coupled to secondary metabolism	Rok7B7, DasR, CprA, GlcNAc-6-P, Glycerol-3-P	Secondary metabolite gene clusters, amino-sugar metabolism (*nagE*), glycolysis gatekeeping	[[29], [30], [31]]

### 2.1 Transport and carbon sensing

CCR begins at the level of carbon entry, where uptake systems function as the first regulatory layer by converting nutrient flux into intracellular signals. In *E. coli*, this role is performed by the glucose-specific phosphotransferase system (Fig. 1A). The phosphorylation state of EIIA^Glc^, determined by the PEP-dependent phosphorelay through EI, HPr, and EIICB^Glc^, serves as a primary signal [1]. During glucose transport, dephosphorylated EIIA^Glc^ accumulates and inhibits non-PTS transporters such as LacY (lactose), MelB (melibiose), and GatABC (galactitol) via direct protein-protein interactions, thereby reducing inducer entry and delaying activation of alternative pathways (i.e. inducer exclusion) [11,[32], [33], [34]]. Meanwhile, dephosphorylated EIIA^Glc^ reduces adenylate cyclase (AC, CyaA) activity, decreasing intracellular cAMP levels (approximately 55–150 nM under glucose-rich vs 200–500 nM or higher under poor carbon sources), which in turn reduces CRP-dependent transcription [1,[35], [36], [37]]. Transport activity is thus converted immediately into a global regulatory signal.

Other bacteria rely less on inducer exclusion and more on intracellular metabolite-responsive modules. In Firmicutes, the bifunctional HPr kinase/phosphorylase (HPrK/P) modulates Ser46 phosphorylation of HPr in response to intracellular levels of ATP and fructose-1,6-bisphosphate (FBP), linking CCR strength to glycolytic flux and energetic state rather than glucose detection alone [1,8,38]. In *Pseudomonas aeruginosa* and *P. putida*, the σ^54^-dependent CbrA-CbrB two-component system senses metabolic status and induces antagonistic sRNAs that tune translational repression [3,16,17]. Actinobacterial systems such as *Corynebacterium glutamicum* similarly lack classical PTS-dependent inducer exclusion and instead integrate carbon status through transcriptional factors such as the cAMP-responsive regulator GlxR and the DeoR-family repressor SugR [3,21,22,39]. Despite mechanistic diversity, a common principle exists, CCR consistently operates through the first layer that converts nutrient uptake and metabolite levels into regulatory information [1,3]. Unlike bacterial PTS systems, fungal CCR lacks a single defined glucose-sensing phosphorelay. Instead, glucose availability is translated into CreA/Cre1 activity through incompletely resolved intracellular carbon-sensing mechanisms.

### 2.2 Transcriptional control and regulon architecture

CCR is typically operated at the transcriptional level, forming the second regulatory layer that reorganises genome-wide expression based on signals generated upstream [1,3]. In *E. coli*, decreased cAMP level under glucose-rich conditions limits the formation of cAMP-CRP complex, leading to reduced expression of operons for alternative carbon utilisation (e.g. *lac*, *mal*, *ara*, *xyl*, *srl*, *fru*, *acs*), as well as subsets of TCA and glyoxylate shunt genes [1,35,36]. Genome-wide ChIP-seq studies have identified 254 CRP-binding promoters, with approximately 75% acting as activation, indicating that CCR reallocates transcriptional capacity across central metabolism, respiration, and biosynthesis to maintain redox and energy balance rather than acting as purely repressive [40]. Additional regulators refine this transcriptional architecture. The glucose-responsive repressor Mlc represses the expression of major glucose PTS transport genes (e.g. *ptsG* and *manXYZ*) under glucose limitation but is sequestered by dephosphorylated EIICB^Glc^ during glucose uptake, enabling rapid derepression of glucose transport independently of cAMP-CRP. Cra (FruR) senses FBP and rebalances fluxes typically by repressing glycolysis while activating gluconeogenic and glyoxylate-shunt functions under non-preferred carbon conditions [1,41].

An analogous but mechanistically distinct system operates in low-GC Gram-positive bacteria. In *B. subtilis*, high glucose availability elevates intracellular FBP levels, which stimulates the kinase activity of HPrK/P, leading to phosphorylation of HPr at Ser46. The resulting HPr(Ser46-P) interacts with CcpA to form an active regulatory complex that binds catabolite-responsive elements(*cre*) and represses genes for non-preferred substrates (e.g., *xyl*, *ara*, *gnt*, *aceBAK*) [8,42]. HprK/P also phosphorylates the HPr-like protein Crh, and both HPr(Ser46-P) and Crh(Ser46-P) can act as CcpA cofactors, making cofactor phosphorylation state a measurable control point for promoter occupancy [8,43]. Structural studies have clarified that HPr-Ser46-P allosterically enhances CcpA DNA-binding affinity, providing a mechanistic bridge from metabolite-controlled phosphorylation to *cre* occupancy [44]. Multi-omics analyses further indicate that approximately 10% of the genome is directly or indirectly regulated by CcpA, extending beyond carbohydrate utilisation pathways (glycolysis, pentose phosphate pathway, gluconeogenesis) to nitrogen assimilation, overflow metabolism (*alsSD*, *ackA*-*pta*), and stress responses [45,46].

Transcriptional CCR regulators frequently extend beyond primary carbon pathways. In the pathogen *Streptococcus pneumoniae*, CcpA shapes virulence gene expression and competence development, demonstrating that nutrient hierarchy can intersect with host-adaptation programmes [26,27,47]. In *Saccharomyces cerevisiae*, Mig1 mediates glucose repression by accumulating in the nucleus and recruiting the Tup1-Ssn6 co-repressor complex to promoters of alternative carbon-utilisation genes (e.g. *GAL1*, *GAL7*, *GAL10*, *MAL11*, *SUC2*), with histone deacetylases reinforcing chromatin silencing (Fig. 1E) [12,48,49]. When glucose declines, Snf1/AMPK becomes activated through phosphorylation by Sak1/Tos3/Elm1, phosphorylating Mig1 and triggering cytosolic relocalisation and derepression [50,51]. A distinct but evolutionarily analogous CCR architecture operates in filamentous fungi, where the C2H2 zinc-finger transcriptional repressor CreA (Cre1 in some species) serves as the central regulator of carbon hierarchy. Unlike bacterial PTS-based systems and the yeast Mig1/Snf1 switch, fungal CCR primarily relies on direct transcriptional repression of alternative carbon utilisation pathways. In *Aspergillus nidulans*, CreA binds conserved promoter motifs (5′-SYGGRG-3′) and represses genes involved in utilisation of non-preferred carbon sources, including ethanol (*alc*), proline (*prn*), and plant polysaccharide degradation pathways (Fig. 1F) [15,29,52]. CreA functions within a hierarchical regulatory network by antagonising pathway-specific activators such as AlcR, XlnR, and AmyR, thereby coordinating the expression of cellulases, hemicellulases, amylases, and other extracellular enzyme systems according to carbon availability. In industrially relevant fungi such as *Trichoderma reesei*, the homologous regulator Cre1 mediates glucose repression of cellulase and hemicellulase genes, directly affecting lignocellulose-degrading enzyme production. In *C*. *glutamicum*, GlxR similarly binds promoters associated with sugar import (*ptsG*, *iolT1*), glycolysis (*pfkA*, *pyk*), TCA cycle (*sdhCAB*, *gltA*), respiratory chain, and amino acid metabolism [21,30]. ChIP-seq showed carbon-source-dependent GlxR occupancy, with distinct binding profiles on glucose vs acetate. SugR adds an additional transcriptional layer by repressing *ptsG* during gluconeogenic growth, and this repression is relieved at high fructose-6-phosphate, thereby tuning glucose uptake to metabolic state [31]. In *Streptomyces*, CCR is coupled to secondary metabolism, including antibiotic, polyketide and siderophore biosynthesis, mediated by nutrient-sensing transcription factors (e.g., DasR, CprA) responding to *N*-acetyl-d-glucosamine 6-phosphate, glycerol-3-phosphate, and other metabolic signals, thereby coordinating primary carbon flux with specialised metabolic modules [[53], [54], [55]]. This illustrates a measurable systems property that CCR can extend into secondary metabolite gene clusters, coordinating primary carbon flux with biosynthetic investment. Collectively, CCR transcription factors define broad regulons integrating central metabolism, stress adaptation, and developmental or virulence traits. Transcriptional reprogramming thus constitutes the structural backbone of CCR.

### 2.3 Post-transcriptional control and translational regulation

RNA-based regulation adds another layer that sharpens repression kinetics and buffers stochastic expression. In *E*. *coli*, the RNA chaperone Hfq coordinates several small regulatory RNAs (e.g. Spot 42, SgrS) that modulate the translation and stability of mRNAs related to alternative carbon uptake and metabolism [56]. Spot 42 is repressed by the cAMP-CRP complex under low-glucose conditions, and it preferentially inhibits CRP-activated transcripts during glucose-rich growth, producing coherent feed-forward regulatory architectures that suppress leaky transcription and delay premature induction of secondary pathways [56]. Furthermore, EIIA^Glc^ interacts with Csr network by modulating the decay of the antagonistic sRNAs CsrB and CsrC via RNase E-targeting protein CsrD, thereby tuning availability of the RNA-binding protein CsrA, a global regulator controlling central metabolism, motility, and biofilm formation [57]. Together, these RNA-mediated mechanisms enable rapid and fine-tuned control of CCR, complementing transcriptional regulation and enhancing system responsiveness.

In *Pseudomonas* spp., CCR primarily operates at the translational level through the Hfq-Crc ribonucleoprotein complex (Fig. 1B) [[58], [59], [60]]. Unlike the classical glucose-centred hierarchy, *Pseudomonads* frequently prioritise organic acids and amino acids over sugars. Under these preferred carbon conditions, Hfq-Crc binds CA-rich motifs in 5′UTRs of target mRNAs (e.g., *amiE*, *benA*, *antABC*, *oprB*), blocking ribosome loading and repressing translation [60]. Upon depletion of preferred carbon sources, the regulatory sRNAs CrcZ and CrcY are strongly induced, titrating Hfq and Crc to relieve translational repression [58,59,61]. The expression of CrcZ/CrcY is controlled by the σ^54^-dependent CbrAB two-component system, which integrates intracellular metabolic status rather than directly sensing individual carbon substrates. Notably, the accumulation of CrcZ/CrcY appears to enable graded modulation of CCR strength, allowing continuous tuning of translational repression rather than a binary switch. Multi-omics analyses have identified hundreds of Hfq-Crc dependent targets, impacting not only carbon source hierarchy but also virulence (e.g. type III secretion), efflux systems, quorum sensing, and amino acid uptake pathways [59,62], demonstrating that CCR in *Pseudomonas* spp. functions as a global resource allocation system integrating metabolism and pathophysiology. Overall, RNA-based control refines output amplitude and timing, thereby enhancing responsiveness and reducing unnecessary protein synthesis during transient nutrient shifts.

### 2.4 Protein turnover and regulatory stability

A final layer of CCR control acts through regulator stability and subcellular localisation, thereby determining the strength and persistence of repression [3]. In filamentous fungi, CreA/CRE1 activity is regulated beyond its transcriptional function through phosphorylation, nuclear-cytoplasmic shuttling, and ubiquitin-mediated turnover. In *A*. *nidulans*, CreA accumulates in the nucleus under glucose-rich conditions to repress target genes but is relocalised and degraded upon carbon depletion [15]. This process is regulated by the CreB-CreC deubiquitination complex, which stabilises CreA by counteracting ubiquitin-dependent degradation; disruption of this pathway reduces CreA stability and alters repression kinetics, whereas defects in ubiquitination can lead to prolonged nuclear retention and sustained repression [15]. Additional components, including ubiquitin ligases and nuclear export machinery, further modulate CreA localisation in response to carbon signals. In *Trichoderma reesei*, Cre1 DNA-binding activity is regulated by phosphorylation, where phosphorylation of Ser241 is required for promoter binding, highlighting a regulatory mechanism distinct from the Snf1-mediated inhibition of the yeast Mig1 orthologue [52]. Transcriptomic analyses in *T*. *reesei* show that the strength of CRE1 regulon scales with growth rate and carbon availability, rather than acting as a binary switch [29]. Together, these findings demonstrate that CCR output is quantitatively tuned through control of regulator half-life and localisation, adding temporal and kinetic regulation beyond transcriptional and RNA-based layers [3].

## 3 Advanced systems approaches extend understanding of CCR mechanisms

CCR is increasingly being dissected through integrated systems-level strategies that convert hierarchical substrate utilisation from a descriptive observation into a quantitative and screenable trait [3]. Recent interactomics have strengthened mechanistic discoveries by mapping regulation across protein-DNA and protein-protein interaction layers. In *E. coli*, integrating ChIP-exo with RNA-seq distinguishes direct regulons of CCR by identifying 49 *in vivo* binding sites associated with 63 candidate transcription units, which are refined to a high-confidence regulation of 35 units, thereby revealing that Cra and CRP antagonistically co-regulated key metabolic nodes [41]. In *Streptococcus pyogenes*, genome-wide CcpA binding mapped 105 occupied loci, revealing direct control over broad transcriptional programs linking carbon metabolism with virulence [63], while CRP ChIP-seq in enteroaggregative *E. coli* uncovered hundreds of CRP binding sites, including virulence-associated targets, highlighting systems-level coupling between nutritional sensing and pathogenicity [64]. Protein-protein interactomics further indicates that CCR outputs can be tuned by regulatory complex assembly; in *P. aeruginosa*, affinity purification-mass spectrometry (AP-MS) identified CrcA (PA1677) as a Crc-interacting factor that disrupts the repressive Crc-Hfq complex, relieving repression and accelerating diauxic transitions on secondary carbon sources [58]. Furthermore, emerging approaches for mapping protein-metabolite and RNA-protein interactions, including limited-proteolysis mass spectrometry (LiP-MS), thermal proteome profiling (TPP), solubility proteome profiling (SPP), co-fractionation MS, RNA immunoprecipitation (RIP), and crosslinking and immunoprecipitation (CLIP), offer scalable strategies to capture ligand-dependent regulation and post-translational control, which may help explain condition-specific CCR behaviours in complex carbon environments. Meanwhile, high-throughput phenomics paired with extracellular metabolomics, especially acoustic droplet ejection MS (ADE-MS) could rapidly quantify substrate depletion kinetics across mixed-carbon conditions and mutant libraries, enabling systematic resolution of substrate hierarchies, co-utilisation patterns, and diauxic lag [65].

Single-cell transcriptomics is beginning to transform the understanding of CCR by demonstrating that repression strength and derepression timing can vary across genetically identical cells. Scalable bacterial platforms (e.g. PETRI-seq and microSPLiT) now enable transcriptome-wide profiling of thousands of individual bacterial cells despite low RNA content and rigid cell walls, revealing metabolic subpopulation structures previously masked by bulk assays [66,67]. In *B. subtilis*, single-cell analysis uncovered pronounced heterogeneity in activation of alternative carbon pathways normally repressed by CcpA. For instance, the *myo*-inositol utilisation operon was induced in only a minority subpopulation [66]. In *E. coli*, high-plex single-cell transcriptomics combined with pseudotime reconstruction during glucose-xylose diauxie revealed that exit from glucose repression is asynchronous and hierarchically structured rather than synchronised [68]. Parallel findings in budding yeast using droplet-based scRNA-seq during glucose-to-maltose shifts revealed bifurcation into transcriptionally distinct fates during the lag phase. One subpopulation induced respiratory genes, stress-response modules, and high-affinity transporters, whereas the other retained a glucose-repressed profile and failed to resume growth [69]. Integration with single-cell ATP measurements further showed that only cells restoring energy balance successfully exited repression, linking CCR heterogeneity directly to metabolic state and growth fate. Together, these studies redefine CCR as a dynamic and distributed state transition governed by subpopulation structure and probabilistic switching, rather than a uniform population-wide regulatory switch.

## 4 Computational modelling of CCR

**Fig. 2:**
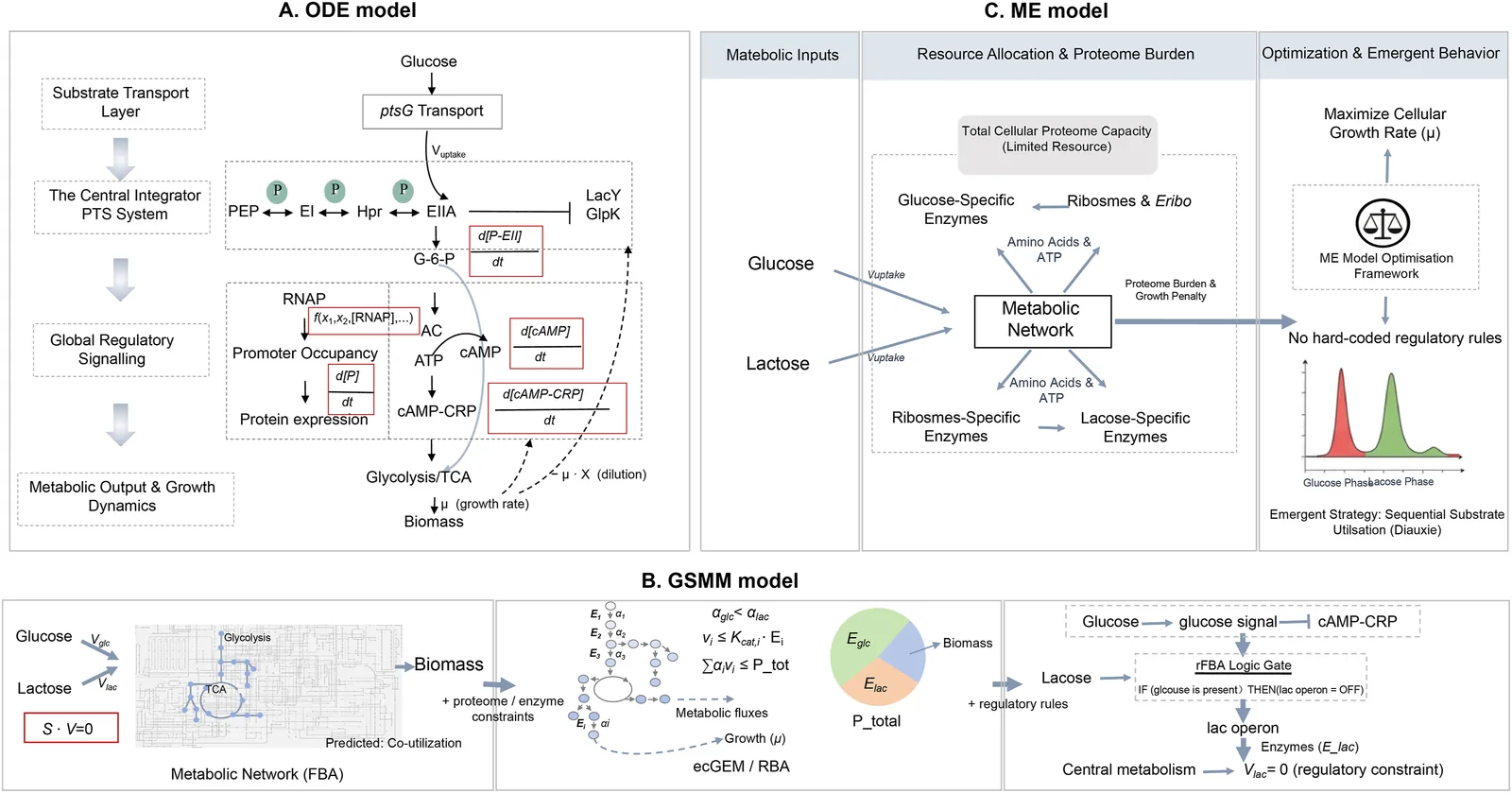
**Multiscale modelling for analysing CCR and diauxic growth.** (**A**) ODE-based kinetic models capture CCR dynamics by linking substrate uptake, PTS phosphorylation, intracellular metabolites, signalling pathways, transcriptional regulation, and cellular growth. These mechanistic models allow diauxic transitions to emerge from time-dependent metabolic and regulatory interactions. (**B**) Genome-scale metabolic models describe metabolism through stoichiometric constraints and flux optimisation. While standard FBA often predicts co-utilisation, extensions such as dynamic FBA, regulatory FBA, enzyme-constrained models, and resource balance analysis incorporate regulation, substrate availability, enzyme capacity, or proteome limits to reproduce sequential substrate use. (**C**) ME models integrate metabolism with gene expression and macromolecular synthesis. By accounting for the synthesis of enzymes, transporters, and ribosome synthesis, they explain CCR-like substrate prioritisation as an emergent outcome of optimal resource allocation rather than imposed regulatory rules.

Computational modelling of CCR has progressed from kinetic ordinary differential equations (ODEs) models that capture signalling and regulatory dynamics, to genome-scale metabolic models (GSMMs, M models) that predict flux distributions under stoichiometric, capacity, and regulatory constraints, and genome-scale metabolism and gene expression (ME) models that further account for the gene expression costs required to sustain metabolic fluxes [10]. A literature-grounded kinetic model of *E. coli* central carbon metabolism exemplifies the mechanistic depth achievable with an ODE approach. The model integrates central metabolism with signalling and transcriptional regulation, containing 27 metabolites, 22 enzymes/PTS proteins, 38 fluxes, 21 gene-expression modules, and 12 biomass-precursor equations, with 351 parameters fitted using a genetic algorithm [70]. Mechanistically, the model links the growth-to-stationary transition to EIIA phosphorylation-driven control of cAMP and CRP-cAMP activity, which rewires central metabolic gene expression. It also quantitatively partitions multilayer regulation, indicating that CRP-cAMP is required to sustain TCA cycle activity, glyoxylate shunt induction, and acetate reutilisation via acetyl-CoA synthetase, whereas Cra-FBP shifts flux between glycolysis and gluconeogenesis/glyoxylate routes. In addition, the model highlights key allosteric control nodes (Pfk, Pyk, Ppc, Fbp, Icl, Mez) and predicts a fast-growing virtual mutant in which loss of FBP activation by PEP increases net glycolytic/TCA flux and ATP production (Fig. 2A) []. It expanded kinetic modelling to full central carbon metabolism with explicit control of multiple transcription factors and non-PTS transport, enabling validation across multiple mutants and conditions. Ensemble diauxie studies further advanced the field by comparing competing ODE, dynamic FBA, and resource-allocation mechanisms, showing that inducer exclusion plus global transcriptional activation are the minimal requirements for robust CCR behaviour [71]. The strength of kinetic models lies in their ability to identify causal control points, reproduce transient behaviour, and evaluate specific regulatory or allosteric perturbations, while the major limitation is parameter uncertainty. Regulatory binding constants, phosphorylation kinetics, enzyme saturation parameters, and intracellular metabolite concentrations are often unavailable or measured under incompatible conditions. As many parameters cannot be uniquely estimated from available data, different parameter sets may reproduce the same observed phenotype while implying different underlying regulatory mechanisms. Agreement with growth curves alone is therefore insufficient; robust validation should also include time-resolved substrate uptake, metabolite concentrations, phosphorylation states, gene expression, and mutant responses. Kinetic models are thus more reliable for well-characterised pathways and conditions, but remain difficult to extend across organisms, environments, or genome-scale networks [70,71].

Constraint-based models trade mechanistic detail for scalability (Fig. 2B). Conventional flux balance analysis (FBA) predicts feasible steady-state flux distributions but generally fails to reproduce diauxic shifts because it assumes instantaneous adaptation and unlimited enzymatic capacity, often favouring simultaneous substrate utilisation. Regulatory FBA (rFBA) partially addresses this limitation by applying Boolean rules that activate or repress reactions according to regulatory states [72]. Genome-scale models of yeast and bacteria integrated global regulators such as cAMP-CRP and Mig1/Snf1, successfully predicting diauxic shifts and growth phenotypes [[73], [74], [75]]. Regulatory constraints can be strengthened further using ChIP-exo, transcriptomics, or experimentally measured uptake rates rather than manually curated Boolean rules. rFBA preserves genome-scale coverage while incorporating known regulatory hierarchies. However, Boolean rules simplify graded, stochastic, and condition-dependent regulation, and transition timing is often imposed rather than predicted. Incomplete and context-specific regulatory networks further limit generalisability. Predictions also depend strongly on the objective function, reaction bounds, maintenance costs, and substrate-uptake constraints. Thus, rFBA is useful for comparing feasible flux states and assessing network-level effects of regulatory perturbations, but is less reliable for predicting dynamic substrate switching without kinetic information or time-resolved experimental constraints.

Enzyme-constrained models, GECKO-type models, resource balance analysis (RBA), and related proteome-allocation frameworks improve physiological realism by limiting flux according to enzyme abundance, turnover number, molecular crowding, or total proteome capacity [13,76]. In these models, diauxic growth can arise because simultaneous expression of multiple catabolic pathways is proteomically inefficient. Optimal allocation therefore favours the substrate that generates the greatest growth benefit per unit enzyme investment, followed by pathway switching when that substrate is depleted. Such models can reproduce classical glucose–lactose diauxie in *E. coli* without explicitly imposing a complete CCR regulatory network [77,78]. Thus, these approaches show substrate hierarchy can emerge from proteome-allocation constraints, enabling analysis of trade-offs among co-utilisation, growth, enzyme burden, and product formation. The accuracy, however depends on parameters such as *k*_cat_, proteome fractions, and condition-specific enzyme usage. As *k*_cat_ are transferred, inferred, or predicted, parameter uncertainty can directly affect predictions of substrate preference. Moreover, proteome-optimal solutions may not reflect regulatory behaviour under stress, fluctuating conditions, or non-growth objectives. Validation should therefore include proteomics, uptake kinetics, enzyme saturation, and flux measurements, not growth rate alone.

Metabolism and gene expression (ME) models provide the most integrated genome-scale representation by coupling metabolic reactions to transcription, translation, ribosome allocation, enzyme synthesis, and macromolecular turnover. A dynamic ME model of *E. coli* shows that diauxic growth, sequential substrate utilisation, and flux reorganisation can emerge from growth optimisation when imposing substantial proteome allocation cost (Fig. 2C) [79]. Importantly, this CCR-like behaviour is not hard-coded, but arises from competition for biosynthetic and translational resources. Compared with enzyme-constrained models, ME models explicitly represent the cost and timing of synthesising the enzymes required for metabolic adaptation. However, ME models require extensive organism-specific information on gene expression, enzyme usage, ribosome activity, macromolecular synthesis, and growth physiology; they are computationally demanding, sensitive to poorly constrained expression parameters, and difficult to validate because substrate preference reflects the combined effects of flux redistribution, protein allocation and growth optimisation [79].

Collectively, modelling studies show that CCR arises from both regulatory control and cellular resource allocation. The choice of modelling framework depends on the biological question: kinetic models resolve dynamic regulatory mechanisms, constraint-based models analyse genome-scale flux distribution, enzyme-constrained models capture proteome-limited substrate hierarchy, and ME models describe emergent behaviours from coupled metabolism and gene expression. Future progress will require integrating these frameworks with time-resolved multi-omics, flux measurements, and high-throughput genetic perturbation data. Coupling models with AI-driven approaches, including machine-learning-guided promoter design, transcription-factor engineering, and predictive identification of regulatory or metabolic targets, could further enable rational optimisation of CCR-controlled mixed-carbon utilisation.

## 5 Adaptive laboratory evolution to overcome CCR constraints

**Fig. 3:**
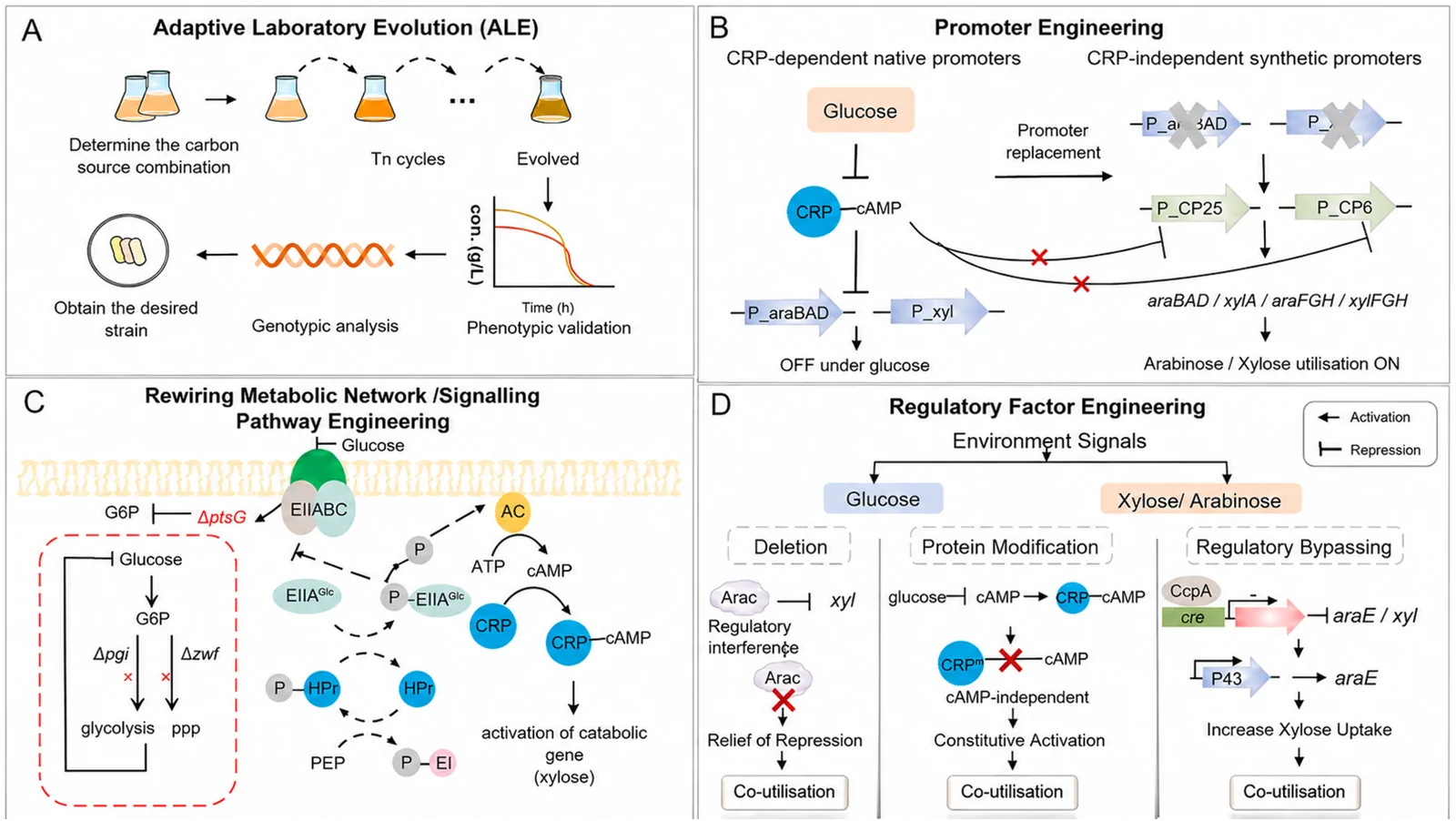
**Engineering strategies to alleviate CCR for simultaneous substrate utilisation.** (**A**) Adaptive Laboratory Evolution (ALE). Selection of evolved strains with relieved CCR through long-term cultivation, followed by genotypic analysis to identify mutations governing the co-utilisation phenotype. (**B**) Promoter engineering. Replacement of CRP-dependent native promoters with synthetic counterparts (e.g., CP25, CP6) to decouple the expression of catabolic and transport operons (*araBAD*, *xylA*) from global carbon-sensing inputs. (**C**) Rewiring Metabolic Network/Signalling pathway engineering. Modification of PTS components (e.g., Δ*ptsG*) to maintain AC activity and intracellular cAMP levels under glucose-rich conditions. (**D**) Regulatory factor engineering. Systematic intervention at the transcription factor level via: (1) Deletion of interfering regulators (e.g., Δ*araC*); (2) Protein Modification using cAMP-independent variants (e.g., CRP^in^); and (3) Regulatory Bypassing through the use of constitutive promoters (e.g., P43) to circumvent CcpA-mediated repression at *cre* sites, thereby enabling robust sugar co-utilisation.

Adaptive laboratory evolution (ALE) improves microbial performance by enriching spontaneous mutations that enhance fitness under defined carbon-selection regimes (Fig. 3A) [80,81]. In CCR studies, ALE is typically designed to force growth on a non-preferred substrate in the presence of glucose or its analogues, thereby selecting strains with faster utilisation of secondary carbon sources, reduced diauxic lag, and improved co-utilisation of mixed substrates, particularly in lignocellulosic feedstocks [82].

In *E. coli*, evolution for improved xylose fermentation often yields mutations in regulatory nodes controlling pentose catabolism. Two substitutions in XylR (R121C and P363S) increased DNA-binding affinity and activated the xylose operon even under glucose repression [83]. Introduction of these mutations into a d-lactate-producing strain enabled consumption of approximately 94% of an equimass glucose-xylose mixture (50 g∙L^−1^ each) and increased product titres by >50% within 96 h [83]. The same XylR variant was later shown to relieve arabinose-induced repression and improve utilisation of glucose-xylose-arabinose mixtures in ethanologenic *E. coli* [84].

Glucose analogue-based ALE provides a direct approach for selecting CCR-attenuated phenotypes. 2-Deoxyglucose (2-DG) is sensed but not fully metabolised; therefore, growth on xylose in the presence of 2-DG enriches mutants that sustain pentose utilisation under glucose-like repression. In *Zymomonas mobilis* 8b, adaptation using 2-DG generated strain #7, which showed improved xylose utilisation in pretreated corn stover liquor and under simultaneous saccharification and fermentation conditions [85]. In the engineered *S. cerevisiae* SR8 strain capable of xylose utilisation, ALE on xylose plus 2-DG produced strains capable of co-utilisation of glucose and xylose. Reverse engineering demonstrated reduced glucose phosphorylation, mediated by mutations in hexokinase genes *HXK1*, *HXK2,* and *GLK1,* was sufficient to confer this phenotype [14]. A complementary forced co-consumption ALE strategy identified mutations in *HXK2*, *RSP5*, and *GAL83*, implicating glucose phosphorylation, hexose transporter activity, and SNF1-associated regulation in mixed-sugar conditions [86].

Similar logic has been extended to non-conventional hosts. In *Kluyveromyces marxianus*, 2-DG-directed evolution yielded the thermotolerant SBK1 mutant, which co-fermented 40 g∙L^−1^ glucose and 28 g∙L^−1^ xylose at 40°C, accompanied by altered GLK1 activity [87]. A subsequent KDH1 mutant further associated CCR mitigation with upregulation of gluconeogenesis and tricarboxylic acid cycle genes [88]. In *Yarrowia lipolytica*, 64-day ALE on xylose plus 2-DG generated a glucose-xylose co-metabolising strain with increased expression of xylose transporter genes and mutations in a glucose transporter and hexokinase [89]. In thermophilic *Parageobacillus thermoglucosidasius*, xylose plus 2-DG evolution relieved CCR and revealed mutations in the PTS components PtsI/PtsG, the ribose operon repressor RbsR, and adenine phosphoribosyltransferase APRT [90]. In *Spathaspora passalidarum* and *Scheffersomyces stipitis*, 2-DG-based evolution improved glucose–xylose co-assimilation and identified an Hxt2.4 transporter variant as a potential engineering target, suggesting a functional alleviation of catabolite repression despite less well-characterised regulatory mechanisms [91].

Overall, ALE studies indicate that adaptation to mixed-carbon environments frequently involves mutations affecting (i) regulatory circuits governing secondary carbon metabolism, (ii) transport systems that determine substrate accessibility, and (iii) glucose sensing or phosphorylation steps. Rather than simply abolishing CCR, these adaptations rebalance substrate uptake, phosphorylation, and central-carbon flux, thereby reducing the competitive dominance of preferred carbon sources and enabling more efficient utilisation of pentoses and other secondary substrates [80,81]. However, ALE-derived phenotypes are highly context-dependent, as evolutionary outcomes are shaped by host backgrounds, carbon-source combinations, selection pressures, and cultivation regimes; therefore, the reported improvements should be interpreted within the experimental context rather than compared directly across studies. In addition, ALE trajectories are often stochastic, and beneficial phenotypes may involve trade-offs between growth, production, stress tolerance, and metabolic efficiency (Table 2). Genome resequencing, reverse engineering, and physiological validation are usually essential to identify causal mutations and translate ALE-derived adaptations into defined engineering strategies [14,[81], [82], [83], [84], [85], [86], [87], [88], [89], [90], [91]].

**Table 2:** Comparative summary of CCR engineering strategies and applications.

Strategy	Engineering principles and representative examples	Reported outcomes	Advantages	Limitations and trade-offs	Key unresolved challenges	References
Adaptive laboratory evolution	Untargeted selection of spontaneous mutations under mixed-sugar or inhibitor-based pressure, with resequencing to identify causal alleles. Examples include *xylR* variants in *E. coli*, 2-DG counter-selection in *Z. mobilis,* and evolved xylose-utilising yeasts.	Diauxic lag shortened or abolished (2 h to 0 h); secondary-sugar uptake increased 1.65–3.6 fold; growth rate increased 2–4 fold. Causal mutations map predominantly to transporters, sugar-specific regulators and global regulators.	Requires no prior mechanistic knowledge; samples epistatic and non-intuitive solutions inaccessible to rational design; yields genetically stable strains.	Trajectories are stochastic and poorly reproducible; genotype-phenotype relationships frequently remain unresolved; selection optimises fitness rather than product formation, such that adaptations may come at the cost of titre or robustness.	Deconvoluting causal from hitchhiking mutations in multi-mutant genomes; predicting and constraining evolutionary trajectories *a priori*; designing selection regimes that couple fitness to product formation rather than growth alone.	[14,[80], [81], [82], [83], [84], [85], [86], [87], [88], [89], [90], [91]]
Carbon sensing and transporter engineering	Rewiring of carbon perception and uptake. Examples include PTS modifications in *E. coli* (*ΔptsG*, *Δcrr*, *ΔptsI*), strengthening PTS-independent glucose uptake via GalP/MglABC, heteroexpression of Glf/Glk, transport engineering via XylE or related permeases.	Mixed-sugar co-utilisation is achieved; secondary-substrate uptake rates are enhanced two fold and the diauxic lag is compressed by 40-fold. PTS-null derivatives generally require compensatory glucose-uptake modules to restore near-parental growth.	Acts at the causal step where repression originates in transport hierarchy or PTS-derived signalling, notably the phosphorylation state of EIIA^Glc^ and consequent cAMP-CRP activation; interventions are modular and transferable across enteric hosts.	Loss of PTS reduces glucose uptake efficiency and perturbs PEP-dependent phosphorylation and PEP/pyruvate balance; import capacity may exceed downstream catabolic capacity, causing intermediate accumulation and protein burden.	Balancing uptake rate against downstream catabolic capacity without incurring overflow metabolism; engineering transporters with defined, non-overlapping substrate specificity; extending PTS-centred design rules to non-enteric and eukaryotic hosts lacking PTS.	[14,41,[92], [93], [94], [95]]
Regulatory-factor engineering	Modification of global or pathway-specific regulators governing the repression hierarchy, including CRP and cAMP-independent CRP variants in *E. coli*, CcpA together with HPr-Ser46-P in *B. subtilis* and related Firmicutes, Crc/Hfq-mediated post-transcriptional repression in *Pseudomonas* spp., and AraR, XylR, Mig1/Snf1 and Cre1/CreA in fungal hosts.	Simultaneous multi-substrate utilisation achieved; derepression of multiple uptake and catabolic modules. Pathway-specific regulator modification has been reported to improve xylose-supported growth by up to approximately 2.5-fold; CRP variants restore catabolic gene expression independently of intracellular cAMP.	Coordinates transport, catabolic and regulatory modules through a limited number of genetic modifications; particularly efficient where a small set of master regulators governs an extensive regulon.	Markedly pleiotropic, as global regulators additionally govern stress adaptation, motility, virulence and central metabolism; consequently, deletion frequently impairs fitness and robustness. Complete knockouts may be lethal or severely growth-limiting, favouring point mutations or tuned expression over deletion.	Separating catabolic derepression from the stress- and fitness-related functions of the same regulator; incomplete mapping of CCR regulons in non-model and industrial hosts; limited understanding of post-transcriptional and metabolite-level regulatory layers (e.g. Crc/Hfq, inducer exclusion) that persist after transcriptional derepression.	[75,[96], [97], [98], [99], [100], [101]]
Promoter engineering	*Cis*-acting modifications that decouple target genes from repression, comprising replacement of CCR-sensitive promoters, deletion or mutation of *cre* and Cre1/CreA-binding motifs, deployment of constitutive or synthetic promoters and construction of synthetic CCR-responsive promoter libraries. Reported applications include promoter replacement within the xylose and arabinose operons.	Target-gene expression is derepressed in the presence of glucose (6.4–12.5-fold increase in transcript or protein level); graded and tunable expression facilitates pathway balancing without incurring the growth penalties associated with loss of global regulators.	Modular, tunable and minimally invasive; global carbon regulation remains intact; readily combined with pathway-level optimisation.	Transcriptional derepression does not reliably translate into increased flux, since post-transcriptional repression (e.g. Crc/Hfq), inducer exclusion and enzyme capacity may remain limiting; overexpression imposes protein burden and generates downstream bottlenecks; promoter performance is strongly context- and host-dependent.	The persistent gap between transcript abundance and metabolic flux; lack of promoters with predictable, portable strength across hosts and growth conditions; absence of design rules linking promoter output to the enzyme level actually required to relieve a given bottleneck.	[75,96,[102], [103], [104], [105]]
Metabolic network rewiring	Redirection of intracellular carbon allocation downstream of uptake, such as blocking G6P flux via Δ*pgi*/Δ*zwf* in *E. coli*, coordinated xylose transport and PPP optimisation in *C. glutamicum*, and *HXK2*/*SNF1*–*MIG1*-related rewiring in *S. cerevisiae*	Carbon redistribution and mixed-sugar conversion are improved (9–18-fold); precursor and cofactor (NADPH) availability is increased (4.48 fold); the dominance of glucose over co-substrate flux is reduced.	Constitutes the sole strategy that couples CCR alleviation directly to product-forming flux, and therefore addresses cases in which co-utilisation has been achieved but does not translate into titre.	Perturbs biomass formation, ATP yield, redox balance and precursor supply; enhanced co-utilisation is frequently obtained at the expense of growth rate or overall yield; outcomes are strongly strain- and condition-dependent.	Quantitatively predicting the growth–yield trade-off before strain construction; limited accuracy of genome-scale models in capturing regulatory and enzyme-capacity constraints under mixed-substrate conditions; scarcity of^13^C-flux data resolving carbon partitioning between co-consumed substrates.	[[106], [107], [108], [109], [110]]

## 6 Engineering regulatory networks

Engineering regulatory networks aims to relieve CCR and promote efficient utilisation of mixed carbon sources [96,111]. These strategies include engineering (i) signalling pathways and transporters, (ii) regulatory factors, and (iii) promoters. These strategies differ substantially in their regulatory scope, engineering complexity, physiological burden, and suitability for different industrial objectives.

### 6.1 Engineering signalling pathways and transporters

Targeting upstream carbon sensing, glucose phosphorylation and transporters provides effective routes to relieve CCR. In bacteria such as *E. coli*, disruption of the glucose-specific PTS permease gene *ptsG* is a widely used strategy (Fig. 3C), which decouples glucose transport from PTS-mediated signalling, restores intracellular cAMP levels, and enhances CRP-dependent activation of pathways for secondary substrates such as xylose, thereby enabling improved sugar co-utilisation [92]. Overexpression of endogenous activator XylR synergistically increased glucose-xylose co-utilisation [92]. In PTS-deficient strains, glucose uptake is re-established through alternative transport and phosphorylation routes, including upregulating endogenous systems such as GalP (proton symporter) and MglABC (ABC transporter) via mutations in GalR, heterologous expression of Glf (glucose transporter) and Glk (kinase), overexpression of high-affinity transporters (e.g. XylE), and engineering of outer membrane porins to enhance sugar permeability. In Gram-positive bacteria, CCR signalling is primarily mediated by HPrK/P. Together, these interventions are frequently highlighted as effective levers for enhancing mixed-sugar fermentation in lignocellulosic bioprocesses [41,93].

In yeasts, signalling-level engineering has focused mainly on glucose phosphorylation and extracellular glucose sensing. In the engineered xylose-metabolising *S. cerevisiae* SR8 strain, expressing the heterologous xylose-utilisation genes *XYL1, XYL2*, and *XYL3*, reducing glucose phosphorylation through mutations or controlled expression of *HXK2*/*HXK1*/*GLK1* switched cells from sequential to simultaneous consumption of glucose with xylose or galactose, demonstrating that intracellular glucose signalling can be tuned directly for co-utilisation [14]. In *Kluyveromyces marxianus*, engineering of KmHXK1/KmSNF1 and, later, the KmSNF3-KmMth1 sensor/receptor pathway generated thermotolerant strains that were derepressed for xylose use while retaining useful glucose assimilation, and these chassis were further developed for xylitol production from hydrolysates [94]. Related transporter-sensor rewiring has also been demonstrated in *Ogataea polymorpha*, where overexpression of the glucose-sensor homologue HXS1 improved ethanol formation and a higher-xylose-affinity HXT1 transporter enabled synchronous glucose-xylose utilisation for high-titre production of free fatty acids and 3-hydroxypropionic acid [95].

Taken together, engineering carbon sensing and transporter systems provides a direct strategy to relieve CCR. These approaches are particularly effective when CCR is mainly driven by PTS-mediated signalling, glucose phosphorylation, extracellular glucose sensing, or transporter-mediated substrate exclusion [41,[92], [93], [94], [95]]. However, disrupting carbon preference at the uptake or sensing level can generate substantial physiological trade-offs. For instance, weakening PTS signalling may compromise glucose uptake efficiency and PEP-dependent metabolic balance, whereas increasing transporter capacity can increase membrane and protein burdens or create mismatches between substrate import and downstream catabolism (Table 2). Future efforts should therefore move beyond simply eliminating carbon hierarchy towards quantitatively balancing signal attenuation, transport capacity, and intracellular flux allocation. Extending these design principles beyond PTS-dependent bacteria and developing transport systems with predictable specificity and minimal metabolic burden remain key challenges for constructing robust co-utilising strains for lignocellulosic and other mixed-substrate bioprocesses.

### 6.2 Engineering regulatory factors

Regulatory factor engineering focuses on modifying the abundance, activity, or DNA-binding regulatory functions of factors that implement CCR at the gene-expression level [96]. In *E. coli*, AraC acts as the master regulator of arabinose metabolism and contributes to hierarchical carbon utilisation involving pentoses. Deletion of *araC* (Fig. 3D left panel) has been associated with enhanced simultaneous utilisation of glucose and xylose, likely due to reduced regulatory interference within pentose metabolism networks under mixed-carbon conditions [75]. In *Klebsiella oxytoca*, expression of a cAMP-independent CRP variant (CRPin) (Fig. 3D middle panel) sustained transcriptional activation of xylose catabolic genes even when cAMP levels were low, thereby enabling simultaneous glucose-xylose utilisation and substantially alleviating glucose repression at the transcriptional level [97].

In low-GC Gram-positive bacteria, CcpA dominates CCR [98]; engineering strategies often focus on bypassing rather than directly modifying the regulator. In *B. subtilis*, overexpression of the broad-specificity sugar permease AraE from a strong constitutive promoter (P43) increased xylose uptake and supported acetoin production from glucose-xylose mixtures (Fig. 3D right panel), demonstrating that targeted transcriptional rewiring can alleviate CCR-imposed limitations without perturbing the global regulatory network [99]. In *Clostridium acetobutylicum*, disruption of *ccpA* abolished glucose repression of xylose use and enabled simultaneous glucose-xylose fermentation while maintaining industrially relevant solvent titres. However, because full loss of CcpA causes pleiotropic effects, subsequent studies moved to fine-tuning (e.g. CcpA V302N mutation) to alleviate CCR with less impact on overall strain performance. Pathway-specific repressors can also be useful targets. Deletion of *araR* increased Clostridial growth on xylose 2.5-fold, and combined *araR*/*xylR* inactivation enabled co-assimilation of arabinose with glucose [112]. In *Bacillus licheniformis*, structure-guided CcpA DNA-binding mutants (e.g. K31A and I42A) weakened repression of xylose in the presence of glucose, illustrating that modulation of regulator–DNA interactions can rebalance carbon hierarchy without fully dismantling global control.

In *P*. *putida* M2, glucose exerts much stronger repression on aromatic catabolic proteins than xylose. CRISPRi-mediated knockdown of *crc* significantly accelerated growth and substrate uptake during glucose–aromatic co-feeding, while sRNA profiling showed that CCR strength is associated with condition-dependent expression of CrcY/CrcZ-like sRNAs [100,101]. These findings underscore translational regulation as another critical control layer and a key engineering target for alleviating CCR in *Pseudomonas*.

Collectively, regulatory factor engineering targets the transcriptional or post-transcriptional nodes that integrate carbon hierarchy into pathway regulation (Table 2). Compared with signalling- or transporter-level interventions, it enables broader CCR modulation by coordinating multiple uptake and catabolic pathways [[97], [98], [99],^112^]. However, the broad regulatory scope of CRP, CcpA, and Crc/Hfq also creates substantial challenges, as these regulators control central metabolism, stress responses, and cellular fitness beyond CCR. Consequently, disruption to these regulators may compromise growth robustness, and future engineering should move towards precision modulation through structure-guided mutations, tunable expression, and context-dependent regulation to decouple CCR control from essential physiological functions [[97], [98], [99], [100], [101],^112^].

### 6.3 Engineering promoters

Promoter engineering can alleviate CCR-associated effects by decoupling target genes from native regulatory inputs, enforcing transcriptional programmes that are less dependent on carbon sensing and global regulatory states [96]. In *Escherichia coli*, native promoters of arabinose and xylose operons are tightly controlled by the cAMP-CRP system; when glucose is present, reduced CRP activity suppresses their expression and limits co-utilisation [102,111]. Replacing these native promoters with synthetic constitutive promoters provides a robust and predictable solution. In an engineered *E. coli* strain capable of simultaneous glucose-xylose utilisation, synthetic promoters such as CP6 and CP25 were used to replace native promoters controlling sugar uptake and catabolism (Fig. 3B) [75]. These promoters drove constitutive expression of key pentose transport and catabolic operons, including *araBAD*, *araFGH*, *araE*, *xylAB*, and *xylFGH*, thereby maintaining pathway activity regardless of glucose availability and alleviating CCR at the level of transcription initiation.

Beyond complete replacement with constitutive promoters, targeted mutagenesis of regulatory elements provides a precise strategy to relieve CCR. In Gram-positive bacteria such as *B. subtilis*, mutation of catabolite-responsive element (*cre*) sites disrupts binding of the global regulator CcpA, thereby alleviating CCR while preserving native promoter dynamics [103]. Similarly, in *T. reesei*, deletion or modification of Cre1-binding sites in cellulase gene promoters enables transcription even under glucose-repressing conditions [104]. Compared to global regulatory perturbations (e.g., *crp* or *ptsG* mutations), which often result in significant pleiotropic effects and growth defects, promoter-level engineering offers a more localised and predictable approach. Moreover, the use of well-characterised synthetic promoter libraries enables fine-tuning of gene expression, facilitating CCR alleviation while minimising metabolic burden [105].

Promoter engineering provides a local and tunable strategy for CCR alleviation by decoupling defined transport or catabolic genes from trancriptional repression [75,96,[102], [103], [104], [105]]. Compared with global regulator modification, promoter replacement or regulatory-site editing minimises perturbation to the regulatory network while enabling pathway-specific control. However, transcriptional derepression does not necessarily translate into improved carbon conversion, as post-transcriptional regulation, enzyme capacity, and downstream metabolic constraints may limit flux, while excessive expression can impose additional protein burden. Future promoter engineering will require more predictable and portable regulatory elements, together with quantitative design rules linking promoter activity to the enzyme levels required for effective pathway optimisation across different hosts and conditions (Table 2).

## 7 Rewiring metabolic network

Metabolic network rewiring has been widely applied across diverse microbial chassis, including *E*. *coli*, *P*. *putida*, and *S*. *cerevisiae*, to improve co-utilisation of glucose and non-preferred carbon sources. These strategies typically involve coordinated optimisation of transport systems, reconstruction of catabolic pathways, and redistribution of central carbon fluxes, thereby reducing carbon-source hierarchy, improving access to alternative substrates, and supporting more balanced mixed-substrate conversion [106,107].

In *E. coli*, a representative flux-based strategy to attenuate glucose-driven carbon catabolite repression involves blocking glucose-6-phosphate (G6P) entry into central metabolism. The majority of G6P is consumed by phosphoglucose isomerase (*pgi*), feeding glycolysis, and glucose-6-phosphate dehydrogenase (*zwf*), initiating the oxidative pentose phosphate pathway. Simultaneous deletion of *pgi* and *zwf* blocks both routes, preventing glucose from efficiently supporting biomass formation and increasing cellular dependence on secondary substrates such as xylose or arabinose. This strategy was demonstrated in d-glucaric acid production, where the Δ*pgi* and Δ*zwf* strain showed reduced glucose repression and substantially increased product yield under mixed-substrate conditions (Fig. 3C red box) [108].

In addition to restricting glucose flux, redistributing carbon flux towards secondary substrates provides another effective rewiring strategy. In *C. glutamicum*, substrate-informed metabolic engineering enables balanced glucose–xylose co-utilisation through coordinated optimisation of transport, catabolic pathways, and central metabolism. Specifically, introduction of xylose assimilation pathways (*xylA* and *xylB*), enhancement of xylose uptake via transporters such as AraE, and optimisation of pentose phosphate pathway flux collectively improve the processing of xylose-derived carbon. These modifications rebalance intracellular carbon flux distribution and reduce glucose dominance, thereby enabling simultaneous utilisation of mixed sugars [109].

In *S. cerevisiae*, metabolic network rewiring is tightly coupled with glucose signalling regulation, where modifications in HXK2 and the SNF1-MIG1 pathway alter carbon flux distribution and effectively integrate regulatory control with metabolic pathway activity [110].

Collectively, these studies demonstrate that carbon catabolite repression can be mitigated through multi-level metabolic rewiring strategies, including flux restriction, pathway strengthening, and system-level carbon integration.

Metabolic network rewiring provides a product-oriented strategy for CCR alleviation by directly redistributing carbon flux between preferred and non-preferred substrates [[106], [107], [108], [109], [110]]. Compared with other rational engineering strategies, this approach directly links CCR relief with precursor supply, pathway flux and target-product formation, making it particularly valuable when co-utilisation is achieved but carbon allocation remains limiting [[106], [107], [108], [109], [110]]. However, central metabolism is tightly coupled to biomass formation, redox balance, and energy generation; therefore, flux redistribution may improve substrate co-utilisation at the expense of growth or production efficiency (Table 2). Future strategies will require predictive models integrating regulatory and enzyme-capacity constraints, supported by quantitative flux measurements, to better balance growth, yield, and carbon partitioning in mixed-substrate bioprocesses.

## 8 Repurposing CCR as a programmable sensor or control module

CCR is conventionally recognised as a barrier to efficient substrate utilisation, but it is increasingly being repurposed as a sensing and control module. This transition aligns with a broader shift in metabolic engineering towards dynamic flux control strategies that autonomously respond to substrate availability, growth phase and cellular state [113]. A recent study developed a genetically encoded glucose uptake rate biosensor (GURB) in *E. coli* by repurposing the CCR regulator Mlc, whose promoter-binding activity changes with glucose uptake. Biosensors were developed by linking Mlc-responsive promoters to fluorescent reporters, in order to track glucose uptake in real time, with the optimised GURB28 variant showing the strongest performance. Beyond monitoring, GURB was integrated into feedback circuits to dynamically regulate central metabolism and improve production of multiple biochemicals, increasing l-tryptophan to 2.64 g/L (+91%), riboflavin to 691.4 mg/L (+59.4%), and d-lactic acid to 15.8 g/L (+40.6%), while suppressing byproduct accumulation [114].

CCR has also been engineered as a programmable control switch for dynamic gene expression. In *B*. *subtilis*, native CCR was inverted to construct a glucose-inducible system by placing a *cre*-modified promoter (*P*_veg-cre_) upstream of a short-lived TetR repressor (via an LVA degradation tag) that controls T7 RNA polymerase. In glucose, CcpA-mediated repression suppresses TetR, activating target gene expression; in non-preferred carbon sources, TetR blocks expression. This design enables a two-stage fermentation strategy, separating growth and production phases to reduce metabolic burden and prevent production escape. The circuit switches rapidly within 10 min, maintains production capacity over long-term cultivation, and improves yields, providing a cost-effective and pathway-independent control system [115]. More recently, the MICA (Metabolizable Inducer-based Carbon-source Autoinduction) system exploited glucose-mediated CCR as an intrinsic autoinduction timer. The engineered carbon-source-responsive promoters were combined with T7 amplification so that expression remained repressed during glucose consumption and was automatically activated after glucose depletion. By adjusting initial glucose levels, induction timing could be predictably tuned, enabling growth-production decoupling and improving protein and carotenoid production, including β-carotene titres of 244.7 mg/L in a 3-L bioreactor [116]. Together, these studies demonstrate that CCR can be transformed from a global inhibitory mechanism into a programmable regulatory layer for low-cost, dynamic bioprocess control.

Beyond CCR-responsive sensors and switches, emerging synthetic biology strategies increasingly combine CCR engineering with programmable regulation, genome-scale perturbation, and biosensor-guided feedback. Carbon-flux-sensing biosensors extend the CCR sensing logic beyond glucose uptake to other metabolic nodes. For instance, *de novo* designed promoters controlled by the catabolite repressor Cra, which responds to FBP as a real-time proxy for glycolytic flux, have been used to dynamically redirect carbon toward target pathways, improving lycopene titres 50-fold (100.3 mg/L) and pyruvate to 9.66 g/L in *E. coli* [117]. Dynamic circuits can also exploit or bypass CCR to coordinate growth and production, as demonstrated by protease-based switches that alleviated CCR through flux redistribution and glucose-repressed sucrose-responsive promoters used carbon depletion as a growth-to-production switching signal [118]. CRISPR-based approaches further extend CCR engineering toward genome-scale regulation. CRISPRi relieved glucose repression to enable glucose-xylose co-utilisation in *B*. *subtilis*, while engineered CRP has been incorporated into multiplex CRISPRa/i systems for programmable transcriptional control in *E. coli* [119,120]. Future efforts combining CCR regulatory constraints into dynamic models and AI-guided strain engineering may enable predictive optimisation of mixed-carbon utilisation while maintaining growth robustness and product formation.

## 9 Concluding remarks and future perspectives

Although CCR is advantageous as a cellular resource-allocation strategy, CCR-driven hierarchical substrate utilisation remains a process-level challenge for mixed-carbon bioprocessing when rapid and balanced conversion of lignocellulose-derived sugars is required. Despite significant progress in ALE and rational engineering, industrial translation is still constrained by condition-specific and organism-dependent CCR regulation, as well as the difficulty of maintaining balanced carbon uptake, growth and production across heterogeneous substrate mixtures [80,96].

Future progress will depend on shifting CCR engineering from empirical strain improvement to predictive, data-driven design. Advances in high-throughput phenotyping and metabolomics now enable rapid, quantitative characterisation of CCR behaviours (e.g., diauxic growth and mixed-sugar co-consumption) across diverse strain-substrate combinations [121], generating datasets that can be leveraged by AI and machine learning. Rather than identifying single targets in isolation, these approaches can help predict how transporter usage, regulatory hierarchies and intracellular flux partitioning jointly determine co-utilisation phenotypes, thereby improving target prioritisation for relieving CCR without compromising process performance. More importantly, because CCR is inherently dynamic, future computational models should be trained on time-resolved datasets that capture diauxic transitions, partial co-consumption and strain-to-strain variation under realistic sugar combinations.

This need is particularly acute for lignocellulosic biomanufacturing, where commercial success depends not simply on consuming glucose efficiently, but on robust and simultaneous utilisation of mixed substrates [107]. Future CCR research should therefore prioritise phenotypes that maintain coordinated uptake of glucose, xylose and arabinose across variable feed compositions, rather than maximising utilisation of a single preferred carbon source [122,123]. In this setting, high-throughput phenomics, extracellular metabolomics and automated cultivation platforms will be essential for mapping carbon use hierarchies at scale and for identifying genotypes that sustain stable co-utilisation across diverse sugar ratios.

Although this review focuses mainly on single-strain strategies for CCR relief or reprogramming, microbial consortia provide an important complementary strategy for mixed-carbon bioprocessing. Instead of forcing a single chassis to express multiple transport and catabolic systems simultaneously, division of labour allows specialised strains to utilise different substrates or perform distinct production functions, thereby reducing the resource-allocation burden on individual cells. Such consortium-based designs may be particularly valuable when feedstocks contain heterogeneous carbon mixtures or when complete co-utilisation by one strain would compromise growth robustness, proteome allocation or production stability. For instance, a two-member *P*. *putida* consortium was engineered for utilisation of polyethylene terephthalate hydrolysate, in which one strain specialised in terephthalic acid degradation and the other in ethylene glycol metabolism, enabling complete substrate assimilation and improved performance compared with monoculture cultivation [124]. Similarly, a glucose-specialist/xylose-specialist *E. coli* consortium compartmentalised sugar utilisation and converted glucose-xylose mixtures into isobutyl butyrate [125]. Thus, consortium-based carbon partitioning represents a complementary strategy to CCR engineering, particularly for industrial processes involving complex mixed-carbon feedstocks.

An integrated DBTL framework provides a practical route to achieve this. In the design phase, genome-scale modelling and machine learning can nominate combinations of transport, regulatory and pathway-level interventions. In the build and test phases, genome engineering and adaptive evolution can be paired with automated phenotyping to quantify sugar uptake order, co-consumption efficiency and carbon partitioning in large strain libraries. The learn phase can then feed these data back into progressively more predictive models, enabling iterative refinement of CCR-relief strategies specifically for mixed-sugar conversion. Applied systematically, this closed-loop workflow should accelerate the development of microbial cell factories that are not merely CCR-relieved in laboratory media, but are optimised for efficient carbon co-utilisation in lignocellulose-based biomanufacturing.

## CRediT authorship contribution statement

**Mengtian Lu:** Conceptualization, Visualization, Writing – original draft. **Haoran Ding:** Conceptualization, Writing – original draft. **Hongyu Zhang:** Writing – review & editing. **Jianyu Xu:** Writing – review & editing. **Ru Meng:** Writing – review & editing. **Yihan Li:** Writing – review & editing. **Zhidan Zhang:** Writing – review & editing. **Ping Zheng:** Writing – review & editing. **Yan Zhu:** Conceptualization, Funding acquisition, Supervision, Writing – review & editing. **Jibin Sun:** Funding acquisition, Writing – review & editing.

## Declaration of competing interest

The authors declare that they have no known competing financial interests or personal relationships that could have appeared to influence the work reported in this paper.

## References

[1] GöRKE B., StüLKE J. (2008). Carbon catabolite repression in bacteria: many ways to make the Most out of nutrients. Nat Rev Microbiol.

[2] Magasanik B. (1961). Catabolite repression. Cold Spring Harbor Symp Quant Biol.

[3] Sonnleitner E. (2025). A comparative analysis: molecular mechanisms of carbon catabolite repression in bacteria. Annu Rev Microbiol.

[4] Mujtaba M., Fernandes Fraceto L., Fazeli M. (2023). Lignocellulosic biomass from agricultural waste to the circular economy: a review with focus on biofuels, biocomposites and bioplastics. J Clean Prod.

[5] Fatma S., Hameed A., Noman M. (2018). Lignocellulosic biomass: a sustainable bioenergy source for the future. Protein Pept Lett.

[6] Kim J.H., Block D.E., Mills D.A. (2010). Simultaneous consumption of pentose and hexose sugars: an optimal microbial phenotype for efficient fermentation of lignocellulosic biomass. Appl Microbiol Biotechnol.

[7] Wang Y., Zhang Y., Cui Q. (2024). Composition of lignocellulose hydrolysate in different biorefinery strategies: Nutrients and inhibitors. Molecules.

[8] Singh K.D., Schmalisch M.H., StüLKE J. (2008). Carbon catabolite repression in *B**acillus subtilis*: quantitative analysis of repression exerted by different carbon sources. J Bacteriol.

[9] Park H., Mcgill S.L., Arnold A.D. (2020). Pseudomonad reverse carbon catabolite repression, interspecies metabolite exchange, and consortial division of labor. Cell Mol Life Sci.

[10] Kremling A., Geiselmann J., Ropers D. (2015). Understanding carbon catabolite repression in *Escherichia coli* using quantitative models. Trends Microbiol.

[11] Hogema B.M., Arents J.C., Bader R. (1998). Inducer exclusion in *Escherichia coli* by non-PTS substrates: the role of the PEP to pyruvate ratio in determining the phosphorylation state of enzyme IIAGlc. Mol Microbiol.

[12] Kayikci Ö., Nielsen J. (2015). Glucose repression in *Saccharomyces cerevisiae*. FEMS Yeast Res.

[13] SáNCHEZ B.J., Zhang C., Nilsson A. (2017). Improving the phenotype predictions of a yeast genome-scale metabolic model by incorporating enzymatic constraints. Mol Syst Biol.

[14] Lane S., Xu H., Oh E.J. (2018). Glucose repression can be alleviated by reducing glucose phosphorylation rate in *Saccharomyces cerevisiae*. Sci Rep.

[15] Ries L.N., Beattie S.R., Espeso E.A. (2016). Diverse regulation of the CreA carbon catabolite repressor in *Aspergillus nidulans*. Genetics.

[16] Valentini M., GarcíA-MauriñO S.M., PéREZ-MartíNEZ I. (2014). Hierarchical management of carbon sources is regulated similarly by the CbrA/B systems in *Pseudomonas aeruginosa* and *Pseudomonas putida*. Microbiology.

[17] GarcíA-MauriñO S.M., PéREZ-MartíNEZ I., Amador C.I. (2013). Transcriptional activation of the CrcZ and CrcY regulatory RNAs by the CbrB response regulator in *Pseudomonas putida*. Mol Microbiol.

[18] Rojo F. (2010). Carbon catabolite repression in *Pseudomonas*: optimizing metabolic versatility and interactions with the environment. FEMS (Fed Eur Microbiol Soc) Microbiol Rev.

[19] Mcgill S.L., Yung Y., Hunt K.A. (2021). *Pseudomonas aeruginosa* reverse diauxie is a multidimensional, optimized, resource utilization strategy. Sci Rep.

[20] Gralka M., Pollak S., Cordero O.X. (2023). Genome content predicts the carbon catabolic preferences of heterotrophic bacteria. Nat Microbiol.

[21] Park S.Y., Moon M.W., Subhadra B. (2010). Functional characterization of the glxR deletion mutant of *Corynebacterium glutamicum* ATCC 13032: involvement of GlxR in acetate metabolism and carbon catabolite repression. FEMS Microbiol Lett.

[22] Engels V., Lindner S.N., Wendisch V.F. (2008). The global repressor SugR controls expression of genes of glycolysis and of the L-lactate dehydrogenase LdhA in *Corynebacterium glutamicum*. J Bacteriol.

[23] Viana R., Monedero V., Dossonnet V. (2000). Enzyme I and HPr from *Lactobacillus**casei*: their role in sugar transport, carbon catabolite repression and inducer exclusion. Mol Microbiol.

[24] Dossonnet V., Monedero V., Zagorec M. (2000). Phosphorylation of HPr by the bifunctional HPr Kinase/P-ser-HPr phosphatase from *Lactobacillus**casei* controls catabolite repression and inducer exclusion but not inducer expulsion. J Bacteriol.

[25] Muscariello L., Marasco R., DE Felice M. (2001). The functional ccpA gene is required for carbon catabolite repression in *Lactobacillus plantarum*. Appl Environ Microbiol.

[26] Iyer R., Baliga N.S., Camilli A. (2005). Catabolite control protein A (CcpA) contributes to virulence and regulation of sugar metabolism in *Streptococcus pneumoniae*. J Bacteriol.

[27] Zhang Y., Zhang J., Xiao J. (2023). comCDE (Competence) operon is regulated by CcpA in *Streptococcus pneumoniae* D39. Microbiol Spectr.

[28] Fleming E., Lazinski D.W., Camilli A. (2015). Carbon catabolite repression by seryl phosphorylated HPr is essential to *Streptococcus pneumoniae* in carbohydrate-rich environments. Mol Microbiol.

[29] Portnoy T., Margeot A., Linke R. (2011). The CRE1 carbon catabolite repressor of the fungus *Trichoderma reesei*: a master regulator of carbon assimilation. BMC Genom.

[30] Jungwirth B., Sala C., Kohl T.A. (2013). High-resolution detection of DNA binding sites of the global transcriptional regulator GlxR in *Corynebacterium glutamicum*. Microbiology (Read).

[31] Engels V., Wendisch V.F. (2007). The DeoR-type regulator SugR represses expression of ptsG in *Corynebacterium glutamicum*. J Bacteriol.

[32] Hariharan P., Balasubramaniam D., Peterkofsky A. (2015). Thermodynamic mechanism for inhibition of lactose permease by the phosphotransferase protein IIAGlc. Proc Natl Acad Sci U S A.

[33] Hoischen C., Levin J., Pitaknarongphorn S. (1996). Involvement of the central loop of the lactose permease of *Escherichia coli* in its allosteric regulation by the glucose-specific enzyme IIA of the phosphoenolpyruvate-dependent phosphotransferase system. J Bacteriol.

[34] Hogema B.M., Arents J.C., Bader R. (1999). Autoregulation of lactose uptake through the LacY permease by enzyme IIAGlc of the PTS in *Escherichia coli* K-12. Mol Microbiol.

[35] Gabor E., GöHLER A.K., Kosfeld A. (2011). The phosphoenolpyruvate-dependent glucose-phosphotransferase system from *Escherichia coli* K-12 as the center of a network regulating carbohydrate flux in the cell. Eur J Cell Biol.

[36] Escalante A., Salinas Cervantes A., Gosset G. (2012). Current knowledge of the *Escherichia coli* phosphoenolpyruvate-carbohydrate phosphotransferase system: peculiarities of regulation and impact on growth and product formation. Appl Microbiol Biotechnol.

[37] Notley-Mcrobb L., Death A., Ferenci T. (1997). The relationship between external glucose concentration and cAMP levels inside *Escherichia coli*: implications for models of phosphotransferase-mediated regulation of adenylate cyclase. Microbiology (Read).

[38] Nessler S., Fieulaine S., Poncet S. (2003). HPr kinase/phosphorylase, the sensor enzyme of catabolite repression in Gram-positive bacteria: structural aspects of the enzyme and the complex with its protein substrate. J Bacteriol.

[39] Chao H., Zhou N.-Y. (2014). Involvement of the global regulator GlxR in 3-Hydroxybenzoate and gentisate utilization by *Corynebacterium glutamicum*. Appl Environ Microbiol.

[40] Shimada T., Fujita N., Yamamoto K. (2011). Novel roles of cAMP receptor protein (CRP) in regulation of transport and metabolism of carbon sources. PLoS One.

[41] Kim D., Seo S.W., Gao Y. (2018). Systems assessment of transcriptional regulation on central carbon metabolism by Cra and CRP. Nucleic Acids Res.

[42] Deutscher J., Reizer J., Fischer C. (1994). Loss of protein kinase-catalyzed phosphorylation of HPr, a phosphocarrier protein of the phosphotransferase system, by mutation of the ptsH gene confers catabolite repression resistance to several catabolic genes of *Bacillus subtilis*. J Bacteriol.

[43] Presecan-Siedel E., Galinier A., Longin R. (1999). Catabolite regulation of the pta gene as part of carbon flow pathways in *Bacillus subtilis*. J Bacteriol.

[44] Schumacher M.A., Allen G.S., Diel M. (2004). Structural basis for allosteric control of the transcription regulator CcpA by the phosphoprotein HPr-Ser46-P. Cell.

[45] Lulko A.T., Buist G., Kok J. (2007). Transcriptome analysis of temporal regulation of carbon metabolism by CcpA in *Bacillus subtilis* reveals additional target genes. J Mol Microbiol Biotechnol.

[46] Moreno M.S., Schneider B.L., Maile R.R. (2001). Catabolite repression mediated by the CcpA protein in *Bacillus subtilis*: novel modes of regulation revealed by whole-genome analyses. Mol Microbiol.

[47] Carvalho S.M., Kloosterman T.G., Kuipers O.P. (2011). CcpA ensures optimal metabolic fitness of *Streptococcus pneumoniae*. PLoS One.

[48] Turcotte B., Liang X.B., Robert F. (2010). Transcriptional regulation of nonfermentable carbon utilization in budding *yeast*. FEMS Yeast Res.

[49] Treitel M.A., Carlson M. (1995). Repression by SSN6-TUP1 is directed by MIG1, a repressor/activator protein. Proc Natl Acad Sci U S A.

[50] DE V.I.T.M.J., Waddle J.A., Johnston M. (1997). Regulated nuclear translocation of the Mig1 glucose repressor. Mol Biol Cell.

[51] Hedbacker K., Carlson M. (2008). SNF1/AMPK pathways in yeast. Front Biosci.

[52] Cziferszky A., Mach R.L., Kubicek C.P. (2002). Phosphorylation positively regulates DNA binding of the carbon Catabolite repressor Cre1 of *Hypocrea jecorina* (*Trichoderma reesei*). J Biol Chem.

[53] Romero-RodríGUEZ A., Rocha D., Ruiz-VillafáN B. (2017). Carbon catabolite regulation in *S**treptomyces*: new insights and lessons learned. World J Microbiol Biotechnol.

[54] Rigali S., Nothaft H., Noens E.E. (2006). The sugar phosphotransferase system of *Streptomyces coelicolor* is regulated by the GntR-family regulator DasR and links N-acetylglucosamine metabolism to the control of development. Mol Microbiol.

[55] Świątek M.A., Tenconi E., Rigali S. (2012). Functional analysis of the N-acetylglucosamine metabolic genes of *Streptomyces coelicolor* and role in control of development and antibiotic production. J Bacteriol.

[56] Beisel C.L., Storz G. (2011). The base-pairing RNA spot 42 participates in a multioutput feedforward loop to help enact catabolite repression in *Escherichia coli*. Mol Cell.

[57] Leng Y., Vakulskas C.A., Zere T.R. (2016). Regulation of CsrB/C sRNA decay by EIIA(Glc) of the phosphoenolpyruvate: carbohydrate phosphotransferase system. Mol Microbiol.

[58] Sonnleitner E., Bassani F., Cianciulli Sesso A. (2023). Catabolite repression control protein antagonist, a novel player in *Pseudomonas aeruginosa* carbon catabolite repression control. Front Microbiol.

[59] Pusic P., Sonnleitner E., BläSI U. (2021). Specific and global RNA regulators in *Pseudomonas aeruginosa*. Int J Mol Sci.

[60] Sonnleitner E., Wulf A., Campagne S. (2018). Interplay between the catabolite repression control protein Crc, Hfq and RNA in Hfq-dependent translational regulation in *Pseudomonas aeruginosa*. Nucleic Acids Res.

[61] MartíNEZ-Valenzuela M., GuzmáN J., Moreno S. (2018). Expression of the sRNAs CrcZ and CrcY modulate the strength of carbon catabolite repression under diazotrophic or non-diazotrophic growing conditions in *Azotobacter vinelandii*. PLoS One.

[62] Yin L., Gong X., Li Y. (2025). The catabolite repression control protein crc regulates the type III secretion System through the adenylate cyclase CyaB in *Pseudomonas aeruginosa*. Microorganisms.

[63] Debroy S., Aliaga-Tobar V., Galvez G. (2021). Genome-wide analysis of in vivo CcpA binding with and without its key co-factor HPr in the major human pathogen group A *Streptococcus*. Mol Microbiol.

[64] Alhammadi M.M., Hothersall J., Lloyd G.S. (2025). Genome-wide mapping of cAMP receptor protein binding in enteroaggregative *Escherichia coli* reveals targeting of virulence-associated genes. Microbiology.

[65] Zhang Z., Zhang C., Zhang X. (2024). Automated and integrated ultrahigh throughput screening for industrial strain enabled by acoustic-droplet-ejection mass spectrometry. J Pharm Anal.

[66] Kuchina A., Brettner L.M., Paleologu L. (2021). Microbial single-cell RNA sequencing by split-pool barcoding. Science.

[67] Blattman S.B., Jiang W., Oikonomou P. (2020). Prokaryotic single-cell RNA sequencing by in situ combinatorial indexing. Nat Microbiol.

[68] Sarfatis A., Wang Y., Twumasi-Ankrah N. (2025). Highly multiplexed spatial transcriptomics in bacteria. Science.

[69] Jariani A., Vermeersch L., Cerulus B. (2020). A new protocol for single-cell RNA-seq reveals stochastic gene expression during lag phase in budding *yeast*. eLife.

[70] Jahan N., Maeda K., Matsuoka Y. (2016). Development of an accurate kinetic model for the central carbon metabolism of *Escherichia coli*. Microb Cell Fact.

[71] Kremling A., Geiselmann J., Ropers D. (2018). An ensemble of mathematical models showing diauxic growth behaviour. BMC Syst Biol.

[72] Covert M.W., Schilling C.H., Palsson B. (2001). Regulation of gene expression in flux balance models of metabolism. J Theor Biol.

[73] HerrgåRD M.J., Lee B.S., Portnoy V. (2006). Integrated analysis of regulatory and metabolic networks reveals novel regulatory mechanisms in *Saccharomyces cerevisiae*. Genome Res.

[74] Österlund T., Nookaew I., Bordel S. (2013). Mapping condition-dependent regulation of metabolism in yeast through genome-scale modeling. BMC Syst Biol.

[75] Kim S.M., Choi B.Y., Ryu Y.S. (2015). Simultaneous utilization of glucose and xylose via novel mechanisms in engineered *Escherichia coli*. Metab Eng.

[76] Goelzer A., Muntel J., Chubukov V. (2015). Quantitative prediction of genome-wide resource allocation in bacteria. Metab Eng.

[77] Beg Q.K., Vazquez A., Ernst J. (2007). Intracellular crowding defines the mode and sequence of substrate uptake by *Escherichia coli* and constrains its metabolic activity. Proc Natl Acad Sci U S A.

[78] Wang X., Xia K., Yang X. (2019). Growth strategy of microbes on mixed carbon sources. Nat Commun.

[79] Salvy P., Hatzimanikatis V. (2021). Emergence of diauxie as an optimal growth strategy under resource allocation constraints in cellular metabolism. Proc Natl Acad Sci U S A.

[80] Sandberg T.E., Salazar M.J., Weng L.L. (2019). The emergence of adaptive laboratory evolution as an efficient tool for biological discovery and industrial biotechnology. Metab Eng.

[81] Sandberg T.E., Lloyd C.J., Palsson B.O. (2017). Laboratory evolution to alternating substrate environments yields distinct phenotypic and genetic adaptive strategies. Appl Environ Microbiol.

[82] Dev C., Jilani S.B., Yazdani S.S. (2022). Adaptation on xylose improves glucose-xylose co-utilization and ethanol production in a carbon catabolite repression (CCR) compromised ethanologenic strain. Microb Cell Fact.

[83] Sievert C., Nieves L.M., Panyon L.A. (2017). Experimental evolution reveals an effective avenue to release catabolite repression via mutations in XylR. Proc Natl Acad Sci U S A.

[84] Martinez R., Flores A.D., Dufault M.E. (2019). The XylR variant (R121C and P363S) releases arabinose-induced catabolite repression on xylose fermentation and enhances coutilization of lignocellulosic sugar mixtures. Biotechnol Bioeng.

[85] Mohagheghi A., Linger J., Smith H. (2014). Improving xylose utilization by recombinant *Zymomonas**mobilis* strain 8b through adaptation using 2-deoxyglucose. Biotechnol Biofuels.

[86] Papapetridis I., Verhoeven M.D., Wiersma S.J. (2018). Laboratory evolution for forced glucose-xylose co-consumption enables identification of mutations that improve mixed-sugar fermentation by xylose-fermenting *Saccharomyces cerevisiae*. FEMS Yeast Res.

[87] Kim S.B., Kwon D.H., Park J.B. (2019). Alleviation of catabolite repression in *Kluyveromyces marxianus*: the thermotolerant SBK1 mutant simultaneously coferments glucose and xylose. Biotechnol Biofuels.

[88] Kwon D.H., Lee S.H., Lee J.S. (2025). Upregulation of the gluconeogenesis pathway was observed by *Kluyveromyces marxianus* KDH1, mitigating glucose catabolite repression. Food Sci Biotechnol.

[89] Zhou L., Wen Z., Wang Z. (2020). Evolutionary engineering improved d-Glucose/Xylose cofermentation of *Yarrowia lipolytica*. Ind Eng Chem Res.

[90] Liang J., Van Kranenburg R., Bolhuis A. (2022). Removing carbon catabolite repression in *Parageobacillus thermoglucosidasius* DSM 2542. Front Microbiol.

[91] Trichez D., Steindorff A.S., DE Morais JúNIOR W.G. (2023). Identification of traits to improve co-assimilation of glucose and xylose by adaptive evolution of *Spathaspora passalidarum* and *Scheffersomyces stipitis* yeasts. Appl Microbiol Biotechnol.

[92] Jung I.Y., Lee J.W., Min W.K. (2015). Simultaneous conversion of glucose and xylose to 3-hydroxypropionic acid in engineered *Escherichia coli* by modulation of sugar transport and glycerol synthesis. Bioresour Technol.

[93] CarreóN-RodríGUEZ O.E., Gosset G., Escalante A. (2023). Glucose transport in *Escherichia coli*: from basics to transport engineering. Microorganisms.

[94] Hua Y., Wang J., Zhu Y. (2019). Release of glucose repression on xylose utilization in *Kluyveromyces marxianus* to enhance glucose-xylose co-utilization and xylitol production from corncob hydrolysate. Microb Cell Fact.

[95] Semkiv M.V., Ruchala J., Tsaruk A.Y. (2022). The role of hexose transporter-like sensor hxs1 and transcription activator involved in carbohydrate sensing azf1 in xylose and glucose fermentation in the thermotolerant yeast *Ogataea polymorpha*. Microb Cell Fact.

[96] Ma Q., Yi J., Tang Y. (2024). Co-utilization of carbon sources in microorganisms for the bioproduction of chemicals. Biotechnol Adv.

[97] Ji X.J., Nie Z.K., Huang H. (2011). Elimination of carbon catabolite repression in *Klebsiella oxytoca* for efficient 2,3-butanediol production from glucose-xylose mixtures. Appl Microbiol Biotechnol.

[98] Warner J.B., Lolkema J.S. (2003). CcpA-dependent carbon catabolite repression in bacteria. Microbiol Mol Biol Rev.

[99] Chen T., Liu W.X., Fu J. (2013). Engineering *Bacillus subtilis* for acetoin production from glucose and xylose mixtures. J Biotechnol.

[100] Shrestha S., Awasthi D., Chen Y. (2023). Simultaneous carbon catabolite repression governs sugar and aromatic co-utilization in *Pseudomonas putida* M2. Appl Environ Microbiol.

[101] Fonseca P., Moreno R., Rojo F. (2013). *Pseudomonas putida* growing at low temperature shows increased levels of CrcZ and CrcY sRNAs, leading to reduced crc-dependent catabolite repression. Environ Microbiol.

[102] Desai T.A., Rao C.V. (2010). Regulation of arabinose and xylose metabolism in *Escherichia coli*. Appl Environ Microbiol.

[103] Miwa Y., Nakata A., Ogiwara A. (2000). Evaluation and characterization of catabolite-responsive elements (cre) of *Bacillus subtilis*. Nucleic Acids Res.

[104] Zou G., Shi S., Jiang Y. (2012). Construction of a cellulase hyper-expression system in *Trichoderma reesei* by promoter and enzyme engineering. Microb Cell Fact.

[105] Alper H., Fischer C., Nevoigt E. (2005). Tuning genetic control through promoter engineering. Proc Natl Acad Sci U S A.

[106] An N., Chen X., Sheng H. (2021). Rewiring the microbial metabolic network for efficient utilization of mixed carbon sources. J Ind Microbiol Biotechnol.

[107] Wu Y., Shen X., Yuan Q. (2016). Metabolic engineering strategies for Co-Utilization of carbon sources in microbes. Bioengineering.

[108] Shiue E., Brockman I.M., Prather K.L. (2015). Improving product yields on D-glucose in *Escherichia coli* via knockout of pgi and zwf and feeding of supplemental carbon sources. Biotechnol Bioeng.

[109] Mees D.J., Cao P., ThöNES A.K. (2026). Substrate-informed metabolic engineering of *Corynebacterium glutamicum* enables balanced glucose-xylose co-utilization for the valorization of lignocellulosic feedstocks. Metab Eng.

[110] Hou J., Qiu C., Shen Y. (2017). Engineering of *Saccharomyces cerevisiae* for the efficient co-utilization of glucose and xylose. FEMS Yeast Res.

[111] Cirino P.C., Chin J.W., Ingram L.O. (2006). Engineering *Escherichia coli* for xylitol production from glucose-xylose mixtures. Biotechnol Bioeng.

[112] Ren C., Gu Y., Hu S. (2010). Identification and inactivation of pleiotropic regulator CcpA to eliminate glucose repression of xylose utilization in *Clostridium acetobutylicum*. Metab Eng.

[113] Ream M., Prather K.L.J. (2024). Engineered autonomous dynamic regulation of metabolic flux. Nat Rev Bioeng.

[114] Ding D., Zhu Y., Bai D. (2025). Monitoring and dynamically controlling glucose uptake rate and central metabolism. Nat Chem Eng.

[115] Tavares L.F., Ribeiro N.V., Zocca V.F.B. (2023). Preventing production escape using an engineered glucose-inducible genetic circuit. ACS Synth Biol.

[116] Li Y., Li Z., Lin Y. (2026). A metabolizable inducer-based carbon-source autoinduction (MICA) system enables low-cost, high-level protein expression and metabolic control in *Bacillus subtilis*. Metab Eng.

[117] Zhu Y., Gao H., Zhang J. (2023). De novo design of the global transcriptional factor Cra-regulated promoters enables highly sensitive glycolysis flux biosensor for dynamic metabolic control. Microb Biotechnol.

[118] Gao C., Hou J., Xu P. (2019). Programmable biomolecular switches for rewiring flux in *Escherichia coli*. Nat Commun.

[119] Williams T.C., Espinosa M.I., Nielsen L.K. (2015). Dynamic regulation of gene expression using sucrose responsive promoters and RNA interference in *Saccharomyces cerevisiae*. Microb Cell Fact.

[120] Moon Soo Y., Kim Mi R., An N.-Y. (2025). Dual-mode CRISPRa/i for genome-scale metabolic rewiring in *Escherichia coli*. Nucleic Acids Res.

[121] Gurdo N., Volke D.C., Mccloskey D. (2023). Automating the design-build-test-learn cycle towards next-generation bacterial cell factories. N Biotechnol.

[122] Li M., Zhu W., Fan J. (2025). Carbon catabolite repression during the simultaneous utilization of lignocellulose-derived sugars in lactic acid production: influencing factors and mitigation strategies. Environ Res.

[123] Lama S., Thapa L.P., Upadhayaya S.K. (2024). Metabolic engineering in lignocellulose biorefining for high-value chemicals: recent advances, challenges, and outlook for enabling a bioeconomy. Front Ind Microbiol.

[124] Bao T., Qian Y., Xin Y. (2023). Engineering microbial division of labor for plastic upcycling. Nat Commun.

[125] Seo H., Castro G., Trinh C.T. (2024). Engineering a Synthetic *Escherichia coli* Coculture for Compartmentalized de novo Biosynthesis of Isobutyl Butyrate from Mixed Sugars. ACS Synth Biol.

